# *rbpms2* functions in Balbiani body architecture and ovary fate

**DOI:** 10.1371/journal.pgen.1007489

**Published:** 2018-07-05

**Authors:** Odelya H. Kaufman, KathyAnn Lee, Manon Martin, Sophie Rothhämel, Florence L. Marlow

**Affiliations:** 1 Department of Developmental and Molecular Biology; Albert Einstein College of Medicine, Bronx, New York, United States of America; 2 Department of Cell, Developmental and Regenerative Biology Icahn School of Medicine at Mount Sinai, New York, New York, United States of America; 3 Department of Neuroscience. Albert Einstein College of Medicine, Bronx, New York, United States of America; University of Wisconsin - Madison, UNITED STATES

## Abstract

The most prominent developmental regulators in oocytes are RNA-binding proteins (RNAbps) that assemble their targets into ribonucleoprotein granules where they are stored, transported and translationally regulated. RNA-binding protein of multiple splice forms 2, or Rbpms2, interacts with molecules that are essential to reproduction and egg patterning, including *bucky ball*, a key factor for Bb formation. Rbpms2 is localized to germ granules in primordial germ cells (PGCs) and to the Balbiani body (Bb) of oocytes, although the mechanisms regulating Rbpms2 localization to these structures are unknown. Using mutant Rbpms2 proteins, we show that Rbpms2 requires distinct protein domains to localize within germ cells and somatic cells. Accumulation and localization to subcellular compartments in the germline requires an intact RNA binding domain. Whereas in zebrafish somatic blastula cells, the conserved C-terminal domain promotes localization to the bipolar centrosomes/spindle. To investigate Rbpms2 functions, we mutated the duplicated and functionally redundant zebrafish *rbpms2* genes. The gonads of *rbpms2a;2b* (*rbpms2)* mutants initially contain early oocytes, however definitive oogenesis ultimately fails during sexual differentiation and, *rbpms2* mutants develop as fertile males. Unlike other genes that promote oogenesis, failure to maintain oocytes in *rbpms2* mutants was not suppressed by mutation of *Tp53*. These findings reveal a novel and essential role for *rbpms2* in oogenesis. Ultrastructural and immunohistochemical analyses revealed that *rbpms2* is not required for the asymmetric accumulation of mitochondria and Buc protein in oocytes, however its absence resulted in formation of abnormal Buc aggregates and atypical electron-dense cytoplasmic inclusions. Our findings reveal novel and essential roles for *rbpms2* in Buc organization and oocyte differentiation.

## Author summary

Oocyte development relies on posttranscriptional regulation by RNA binding proteins (RNAbps). RNAbps form large multi-molecular structures called RNPs (ribonucleoproteins) that further aggregate into regulatory granules within germ cells. In zebrafish primary oocytes, a large transient RNP aggregate called the Balbiani body (Bb) is essential for localizing patterning molecules and germline determinants within oocytes. RNA-binding protein of multiple splice forms 2, or Rbpms2, localizes to germ granules and the Bb, and interacts with *bucky ball*, a key factor for Bb formation. We show that Rbpms2 requires RNA binding for localization within germ cells, and that the C-term and RRM contribute to Rbpms2 subcellular localization in distinct somatic cell types. To investigate Rbpms2 functions we mutated the duplicated zebrafish *rbpms2* genes. Consistent with redundant functions, *rbpms2a* and *rbpms2b* gene expression overlaps, and single mutants have no discernible phenotypes. Although *rbpms2a;2b* double mutants have cardiac phenotypes, those that reach adulthood are exclusively fertile males. Genetic analysis shows that *rbpms2* mutant oocytes are not maintained even when *Tp53*, a regulator of cell death is absent. Initial oocyte polarity is established in *rbpms2* mutants based on asymmetric distribution of Buc protein and mitochondria; however, abnormal Buc structures and atypical cytoplasmic inclusions form. This work reveals independent Rbpms2 functions in promoting Bb integrity, and as a novel regulator of ovary fate.

## Introduction

Two major objectives of oocyte development are to produce haploid gametes through meiosis, and to prepare the ovulated egg for successful fertilization and early embryonic development. Unlike most developmental programs that are regulated by transcription factors, the developmental programs of oocyte maturation, egg fertilization, and early embryonic development take place while the oocyte and early embryonic genomes are transcriptionally silent (reviewed in [1, 2]). During this period, RNA-binding proteins (RNAbps) are the predominant post-transcriptional regulators that coordinate localization and translation of the RNA molecules encoding the proteins that govern processes essential to oogenesis and early embryogenesis.

The RNAbp RNA-binding protein with multiple splicing, RBPMS, family is generally represented by two paralogs in vertebrates, RBPMS and RBPMS2 [3]. The RNA recognition motif of RBPMS family members contains two ribonuclear protein domains, RNP1 and RNP2, which contain the 6–8 residue structural elements which bind to RNA [4–6]. RBPMS proteins associate with poly-adenylated mRNAs *in vitro* [7], and PAR-CLIP followed by RNA sequencing identified the 3’UTR of target RNAs as the primary region to which RBPMS proteins bind (~ 35%), followed by intronic regions (~ 20%) and coding sequence (~10%) [3]. Interestingly, the association with intronic regions suggests that RBPMS proteins can interact with pre-mRNA, and indeed, RBPMS/RBPMS2 can shuttle between nuclear and cytoplasmic fractions [3].

In germ cells, RNAbps associate with RNAs into supramolecular complexes called RNPs (ribonucleoproteins), which further aggregate into granules that are a hallmark feature of primordial germ cells (PGCs), and oocytes of various stages (reviewed in [8, 9]). In primary oocytes, a transient structure called the Balbiani body (Bb) is a single, large, cytoplasmic aggregate of RNPs, scaffolding proteins, and other patterning molecules which indicates the future vegetal pole of the oocyte [10]. The RNAbp RNA-binding protein with multiple splicing (Rbpms), or *hermes* in *Xenopus*, localizes to the Bb of frog and zebrafish oocytes [11, 12], where it interacts with Bucky ball protein (called Velo1 in *Xenopus*), the only vertebrate gene known to be required for Balbiani body formation [13–16]. Zebrafish Rbpms2b also binds to the *bucky ball* transcript, which contains numerous predicted Rbpms2 RNA recognition elements within its introns and 3’UTR [14]. In spite of Rbpms2 localization to the Bb of oocytes and the presence of these important biochemical interactions, the function of Rbpms2 in oocyte development or Bb formation has not been well elucidated.

In this work, we characterized the localization of wild-type and mutant Rbpms2 proteins to cellular RNA granules, including germ granules of PGCs, the Bb of oocytes, and granules within somatic cells. Rbpms2 localization to germ granules and the Bb of oocytes is dependent on its RNA binding domain. In zebrafish somatic cells, this domain is sufficient for granule localization, while the C-term domain promotes association with the bipolar spindle at the expense of granules. In HEK 293 cells, RNA binding is dispensable for granule localization, indicating Rbpms2 uses different domains to achieve its subcellular localization in diverse cell types. To investigate Rbpms2 functions, we generated zebrafish mutants disrupting the duplicated *rbpms2* genes, *rbpms2a* and *rbpms2b* using Crispr-Cas9 mutagenesis. These analyses revealed that Rbpms2 is essential for oocyte development as r*bpms2* double mutants (hereafter *rbpms2* mutants) develop exclusively as fertile males. Zebrafish *rbpms2* mutants have normal germline development until the onset of sexual differentiation, at which time *rbpms2* mutants can initiate, but not complete, oocyte differentiation. Testis differentiation is unimpaired in *rbpms2* mutants. Ultrastructural analysis revealed early oocytes of *rbpms2* mutants can asymmetrically accumulate mitochondria like their normal siblings, but have large cytoplasmic inclusions that were not present in wild-type. Based on the presence but unusual localization of Bucky ball protein in early oocytes of *rbpms2* mutants, we conclude that Rbpms2 is dispensable for Buc translation; however, Rbpms2 seems to be required for structural integrity of Buc aggregates. In addition, Rbpms2 has a Buc-independent function in ovary maintenance. This data reveals a novel and essential role for *rbpms2* in oogenesis and Bucky ball organization within early oocytes.

## Results

### Expression of *rbpms* family genes in zebrafish embryos and oocytes

In *Xenopus*, *hermes* is expressed in maturing oocytes, and *hermes* RNA and protein are both localized to the Balbiani body of primary oocytes [12]. Similarly, in primary zebrafish oocytes, the *Xenopus* anti-Hermes antibody detects a Bb-localized Hermes homolog [11, 15]. Zebrafish have three Hermes homologs encoded in their genome: Rbpms (hereafter Rbpms1 for clarity), and the similar proteins Rbpms2a and Rbpms2b, the products of *rbpms2* gene duplication in zebrafish [17]. Additionally, *Xenopus* Hermes interacts with the Bb-localized Bucky ball homolog Velo1 [16], as do all three protein products of the zebrafish Rbpms family (Fig. S1M in S1 Supporting Information) [14]. Phylogenetic analyses of RBPMS proteins have previously been described [3, 18, 19]; however, none directly compared zebrafish RBPMS proteins with *Xenopus* Hermes. Thus, we compared protein similarity of Hermes and the zebrafish Rbpms family, which revealed that zebrafish Rbpms2a/b proteins clustered more tightly together with Hermes, and thus likely represent the closest *Xenopus* Hermes homolog (Fig. S1A in S1 Supporting Information) [20, 21]. Maternal RNA transcripts for *rbpms1*, *rbpms2a*, and *rbpms2b* were all abundant in early embryonic stages prior to the maternal-zygotic transition, after which their levels were severely reduced (Fig. S1B–E” in S1 Supporting Information). Examination of *rbpms2* transcripts during zebrafish embryogenesis by whole-mount *in situ* hybridization (ISH) revealed that *rbpms2a* and *rbpms2b* were expressed in the embryonic heart, retina and pronephros, an evolutionarily conserved expression pattern similar to that previously reported for *hermes* homologs in other vertebrates, including *Xenopus*, chicken, and mouse (Fig. S1H–K in S1 Supporting Information) [7]. Low levels of *rbpms1* expression were ubiquitous throughout most tissues of 24-48hpf embryos (Fig. S1F–G in S1 Supporting Information).

**Fig 1:**
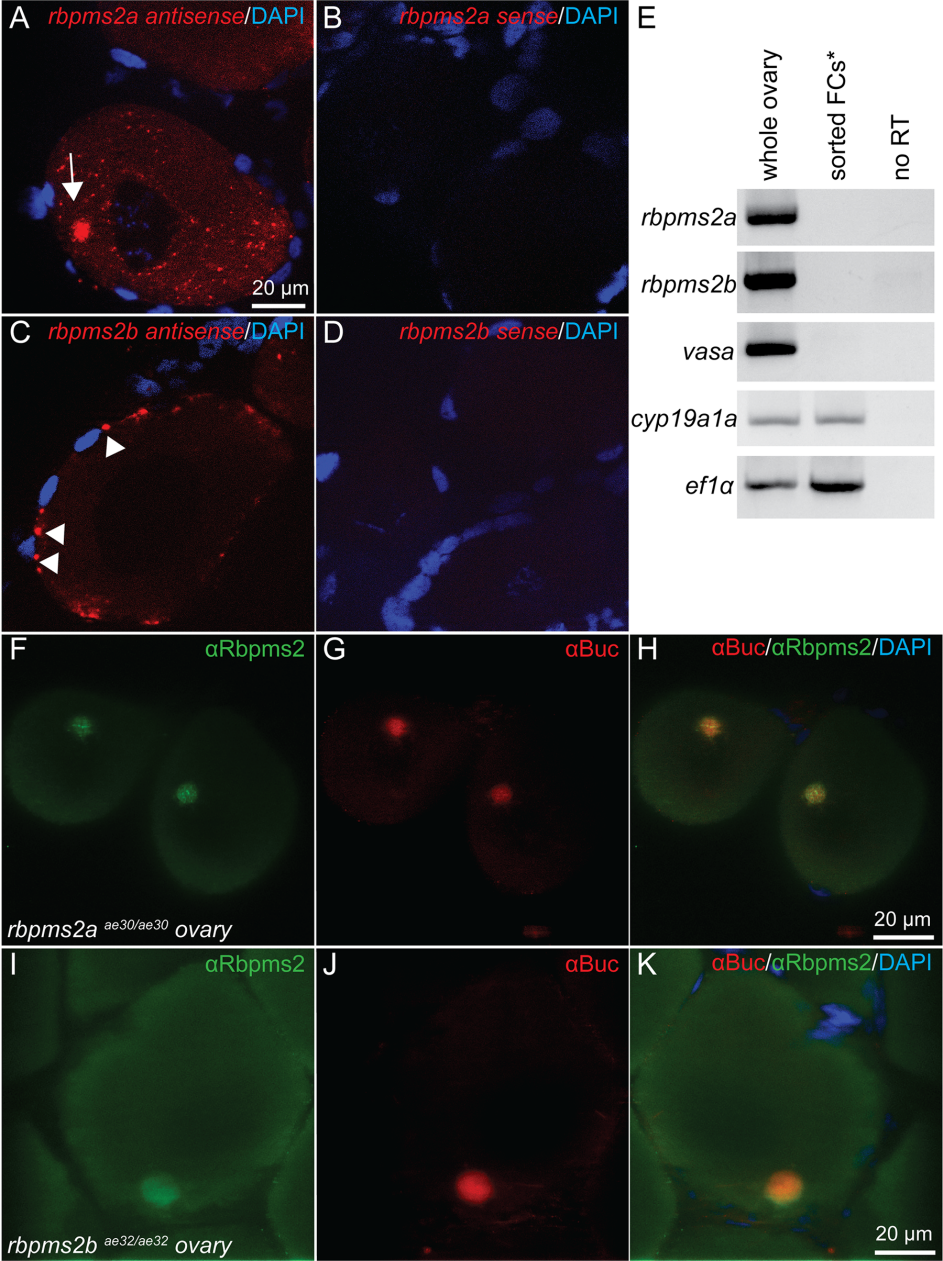
Expression of *rbpms2* in oocytes. Fluorescent *in situ* hybridization displaying Bb localization (arrow) of *rbpms2a* transcripts (A), while *rbpms2b* transcripts are not Bb-localized (C). Sense control probes demonstrate that *rbpms2a* and *rbpms2b* signals are specific (B,D). (E) RT-PCR on FACS-sorted granulosa and theca cells demonstrates that expression of *rbpm2a* and *rbpms2b* is germ cell specific. Antibody staining for Rbpms2 in ovaries mutant for *rbpm2a* (F) or *rbpms2b* (I) suggests both ohnologs are Bb localized where Bucky ball protein also resides (G,H,J,K).

Next we examined *rbpms1* and *rbpms2a/b* expression in zebrafish oocytes. We found that while all three transcripts were expressed in primary oocytes (Fig 1A–1D and Fig. S1L in S1 Supporting Information), only *rbpms2a* transcripts were enriched within the Bb (Fig 1A, arrow). In contrast, *rbpms2b* transcripts appeared to be enriched at the oocyte cortex or within the granulosa cell layer (Fig 1C, arrowheads). *RT-PCR* on FACS-sorted GFP-positive granulosa and theca cells from *Tg[cyp19a1a*:*GFP]* transgenic zebrafish ovaries, revealed no *rbpms2* expression in these cell populations [22] (Fig 1E). However, because Cyp19a1a:GFP is only expressed in granulosa cells of stage II follicles or later [22], we cannot exclude the possibility that *rbpms2* RNAs are transiently expressed in granulosa cells of stage I follicles, then rapidly down-regulated prior to stage II.

Finally, we tested whether both Rbpms2 proteins were expressed in primary oocytes (stage I) and localized to the Bb. We used two commercial antibodies raised against human Rbpms2 which we predicted would recognize both Rbpms2a and Rbpms2b based on protein similarity (Fig. S2A in S1 Supporting Information). Stained ovaries from zebrafish mutants for *rbpms2a* (Fig 1F and Fig. S3A,E in S1 Supporting Information) or *rbpms2b* (Fig 1I, Fig. S3B,F in S1 Supporting Information) both exhibited staining of Rbpms2 in the Bb, likely representing the ohnolog protein, or alternatively a truncated mutant protein (mutants described in next section). The absence of signal in *rbpms2* double mutants indicates antibody specificity for Rbpms2s (Fig. S3C in S1 Supporting Information). Rbpms2 protein in single mutant ovaries is localized to the Bb along with Bucky ball (Fig 1F–1K). Thus, consistent with the expression of *Xenopus* Hermes, we conclude that both Rbpms2a and Rbpms2b proteins are localized to the Bb of zebrafish oocytes.

### Functional redundancy between *rbpms2a* and *rbpms2b* genes

**Fig 2:**
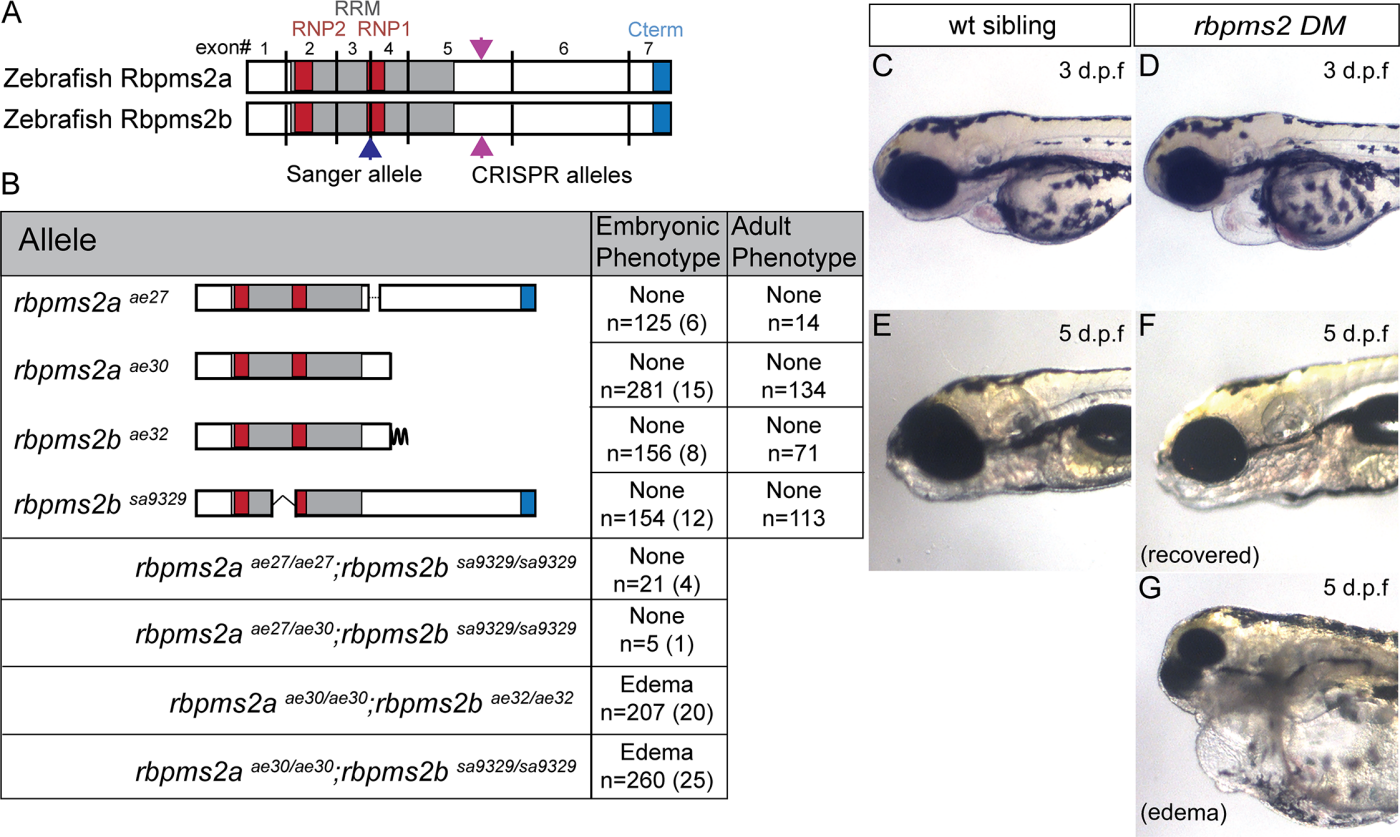
Rbpms2 embryonic phenotype characterization. (A) *rbpms2a* and *rbpms2b* alignment showing extremely similar exon structure, with the pink arrowheads pointing to the Crispr-Cas9 disrupted region of exon 5 in both *rbpms2a* and *rbpms2b*, and the blue arrowhead pointing to the disrupted splice site in *rbpm2b*. (B) Table of germ-line *rbpms2* alleles and predicted protein structure, with single and compound mutant phenotypes represented in the two right columns. In the table, n = number of embryos of that genotype, and the number in parentheses represents independent parental crosses from which those embryos were derived. Representative images of the lateral view of d3 embryos, showing head and cardiac structures of non-mutant sibling (C) and *rbpms2a*^*ae30/ae30*^; *rbpms2b*^*sa9329/sa9329*^ double mutant (D). Representative image of lateral view of d5 embryos, showing head and cardiac structures of non-mutant sibling (E) and recovered (F) and edematous (G) *rbpms2a*^*ae30/ae30*^*; rbpms2b*^*sa9329/sa9329*^ double mutants.

We anticipated that *rbpms2a* and *rbpms2b* might function redundantly based on protein similarity (92% identity) and their comparable gene expression profiles (Fig 1 and Fig. S1 in S1 Supporting Information). Therefore, to study *rbpms2* functions we generated zebrafish mutant lines disrupting both *rbpms2a* and *rbpms2b* using CRISPR/Cas9 mutagenesis [23]. After numerous unsuccessful attempts to mutagenize upstream regions of the *rbpms2a* and *rbpms2b* genes, we ultimately succeeded in mutating analogous regions of exon 5 in *rbpms2a/2b* using CRISPR sites predicted by the web-based ZiFiT targeting program (https://crispr-cas9.com/96/zifit-targeter-crispr-cas9/) (Fig 2A and Fig. S2B,C in S1 Supporting Information). We recovered germline-transmitted mutant alleles for each *rbpms2* gene. Sequencing of genomic regions as well as mutant cDNAs revealed that the isolated mutations were as follows: the *rbpms2a*^*ae27*^ allele had an in-frame deletion of 9 amino acids, the *rbpms2a*^*ae30*^ allele had a 15bp deletion and 17bp insertion resulting in a truncated protein (Fig. S2B in S1 Supporting Information), and the *rbpms2b*^*ae32*^ allele had a 20bp insertion resulting in a truncated protein (Fig. S2C in S1 Supporting Information). Additionally, we characterized a splice-site mutation from the Sanger Institute’s Zebrafish Mutation Project [24], allele *rbpms2b*^*sa9329*^, which was found to contain a T>A point mutation in the 5’ splice site between exon 3 and 4 that causes in-frame skipping of exon 3 and is predicted to partially disrupt RNP1 (Fig. S2C in S1 Supporting Information).

We found that zebrafish embryos homozygous for mutations in *rbpms2a* or *rbpms2b* had no observable morphological differences from their wild-type siblings. Adult fish mutant for a single *rbpms2* gene had no apparent phenotypes, and differentiated into fertile fish of either sex (Fig 2B). Moreover, no overt maternal-effect, paternal-effect or maternal-zygotic phenotypes were observed in the progeny of single mutant adults for *rbpms2* genes (n>20 adult mutants per allele examined). To test the possibility that the *rbpms2* genes were functionally redundant, we made *rbpms2a;rbpms2b* double mutants. At 3 days post fertilization (d3) no phenotypes were observed when a single functional copy of *rbpms2* was present; however, double mutant embryos for the truncation alleles (*rbpms2a*^*ae30/ae30*^*;rbpms2b*^*ae32/ae32*^) or for the *rbpms2a* truncation allele and the *rbpms2b* exon-skipping allele (*rbpms2a*^*ae30/ae30*^*;rbpms2b*^*sa9329/sa9329*^) displayed cardiac edema phenotypes (Fig 2C and 2D). This cardiac phenotype is consistent with the conserved expression of *rbpms2* in the embryonic heart (Fig. S1H–K in S1 Supporting Information)[25, 26], as well as the previously published cardiac phenotypes caused by *hermes/rbpms2* overexpression in *Xenopus* [7, 25, 26]. Consistent with previous findings that demonstrate Rbpms proteins function as dimers [6, 7, 27], we found that zebrafish Rbpms2a and Rbpms2b can both homodimerize, and Rbpm2a can heterodimerize with Rbpms2b (Fig. S6B in S1 Supporting Information). Thus, there is likely no requirement for heterodimers and no difference in Rbpms2a or Rbpms2b homodimer function since complete loss of a single *rbpms2* gene is still sufficient for normal Rbpms2 function.

Approximately half of the embryos with cardiac edema at d3 recovered by d5 and were raised to adulthood (47±18%, based on quantification of 71 total edematous embryos at d3 from 9 parental in-crosses) (Fig 2E–2G). In contrast, no cardiac edema phenotypes were observed in *rbpms2a*^*ae27/ae27*^*;rbpms2b*^*sa9329/sa9329*^ double mutants or *rbpms2a* trans-het;*rbpms2b* double mutants (*rbpms2a*^*ae27/ae30*^*;rbpms2b*^*sa9329/*sa9329^)(Fig 2B). This analysis suggests that the *rbpms2a*^*ae27*^ allele retains sufficient activity to fulfill Rbpms2 functions; whereas, *rbpms2a*^*ae30*^, *rbpms2b*^*ae32*^, and *rbpms2b*^*sa9329*^ are loss-of-function mutations. Therefore, we focused subsequent analyses on the double mutants *rbpms2a*^*ae30/ae30*^*;rbpms2b*^*ae32/ae32*^ or *rbpms2a*^*ae30/ae30*^*;rbpms2b*^*sa9329/sa9329*^, hereafter referred to as *rbpms2* DM (double mutants) or simply *rbpms2* mutants.

### Characterization of *rbpms2* mutant allele stability and activity

**Fig 3:**
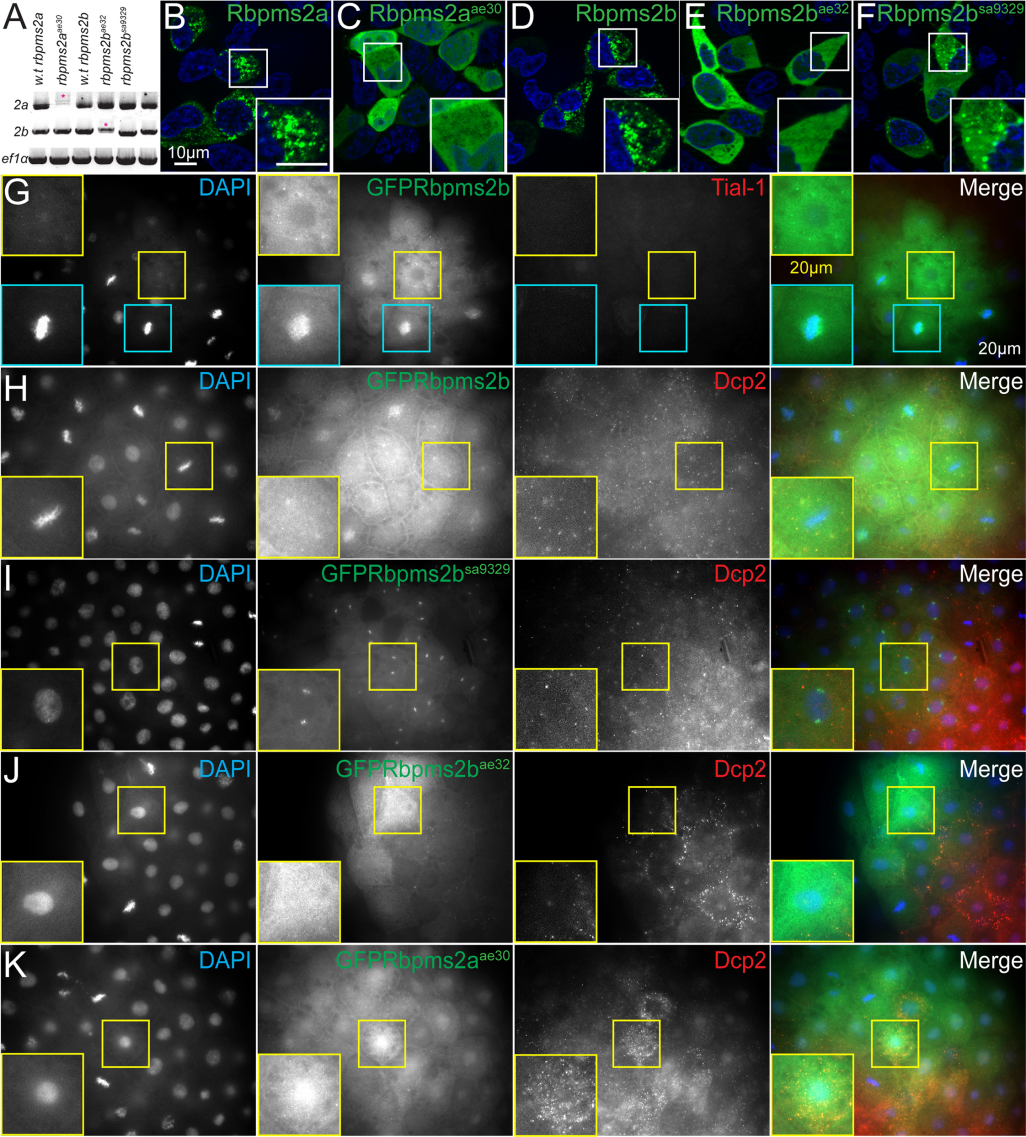
*rbpms2* mutant allele stability and localization activity in somatic cells. (A) RT-PCR to detect *rbpms2a* and *rbpms2b* maternal transcripts. Pink asterisks indicate likely nonsense mediated decay of mutant allele transcripts. (B-F) Wild-type GFP-Rbpms2a/b (B, D) and GFP-Rbpms2b^sa9329^ (F) localize to granules in HEK 293 cells, while GFP-Rbpms2a^ae30^ and GFP-Rbpms2b^ae32^ are not localized. (G-K) Zebrafish blastula cells expressing GFP-Rbpms2 fusions. (G,H) Wild-type GFP-Rbpms2b localizes near the nucleus (H), is apparently associated with the centrosome/spindle in some cells, and (G) is in granules that are not positive for the stress granule marker Tial-1. (H) A subset of GFP-Rbpms2b positive granules are positive for the p-body marker Dcp2 (open arrowheads). (I) GFP-Rbpms2b^sa9329^ localization to the centrosome/spindle but not granules. (J) GFP-Rbpms2b^ae32^ and (K) GFP-Rbpms2a^ae30^ localization. Insets show magnified views of the highlighted cells. Images are representative slices from Z-stacks of sphere stage embryos viewed from the animal pole.

To examine the stability of the mRNA for the different *rbpms2* alleles, we performed RT-PCR on RNA extracted from maternal-zygotic (MZ) mutant embryos at the 4-cell stage. This embryonic stage precedes zygotic genome activation; thus, the contributions of maternal transcripts from *rbpms2a* or *rbpms2b* single mutant mothers can be examined. We found that the *rbpms2a*^*ae30*^ and *rbpms2b*^*ae32*^ transcripts were detectable, but appeared to be less abundant than wild-type, indicative of potential nonsense mediated decay (NMD) (Fig 3A, pink asterisks). This is consistent with descriptions of NMD in zebrafish, which does not typically lead to complete degradation of transcripts containing early stop codons [28]. The finding of NMD further supports the notion that *rbpms2a*^*ae30*^ and *rbpms2b*^*ae32*^ are likely null alleles.

Mammalian RBPMS has previously been studied in HEK293 cells, where it has been demonstrated to localize to stress granules along with poly-adenylated RNAs and the stress granule marker G3BP1 [3]. Therefore, we used this established localization assay to examine the stability and activity of the Rbpms2 proteins encoded by the zebrafish mutant alleles. First, we examined the localization of the wild-type zebrafish Rbpms2 proteins in HEK293 cells using GFP protein fusions. We found that GFP-Rbpms2a and GFP-Rbpms2b were each predominately localized to small punctate structures resembling the previously described stress granules (Fig 3B and 3D). Stable GFP fusion proteins for GFP-Rbpms2a^ae30^ and GFP-Rbpms2b^ae32^ were detected in HEK293 cells, indicating that the mutant RNAs and proteins were sufficiently stable to be visualized in this context; however, in contrast to the punctate localization of the WT proteins, the GFP-Rbpms2a^ae30^ and GFP-Rbpms2b^ae32^ proteins were diffusely cytoplasmic (Fig 3C and 3E). GFP-Rbpms2b^sa9329^ exhibited an intermediate localization phenotype, displaying both diffuse GFP fusion protein as well as localization to somatic cell granules (Fig 3F), suggesting this allele may behave as a hypomorphic reduction-of-function allele. Taken together these data indicate that the C-terminus is required for localization to granules in this somatic cell type.

**Fig 4:**
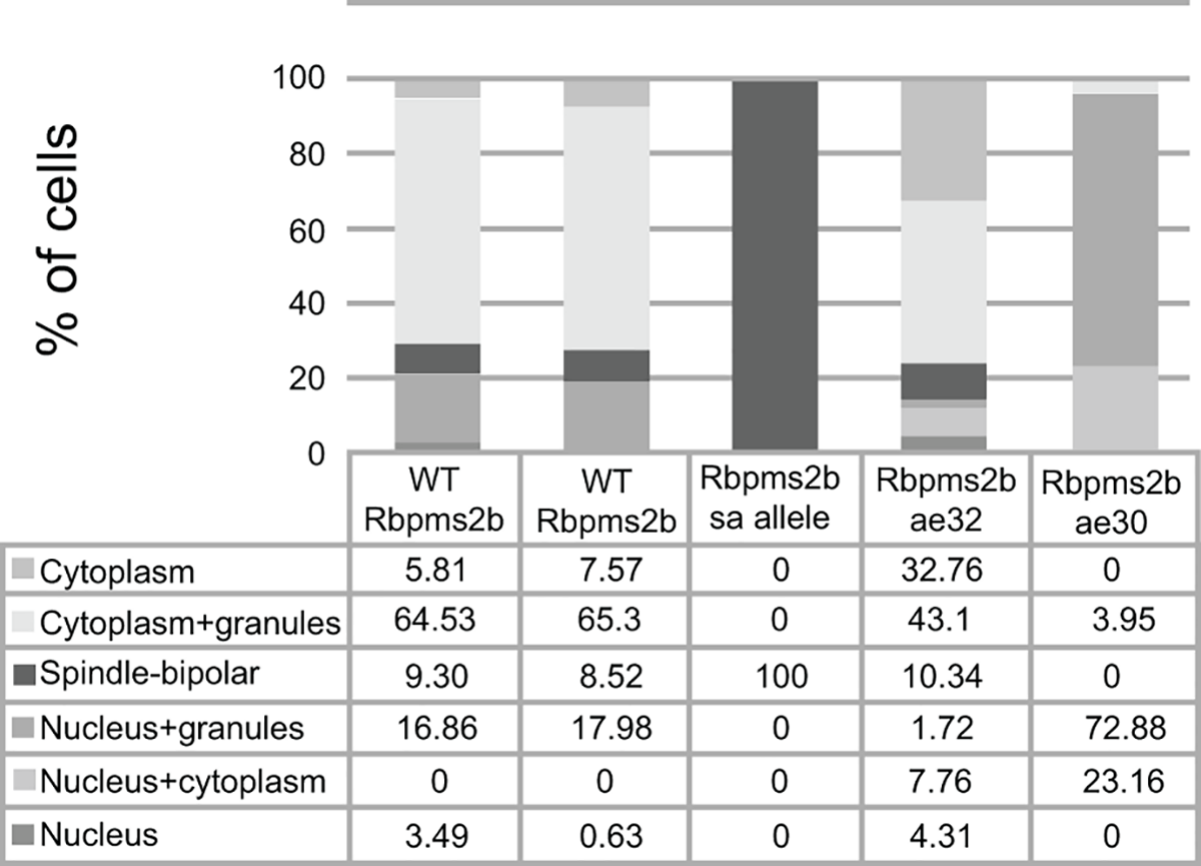
Quantification of fusion protein subcellular localization in zebrafish somatic cells.

Next, we examined the localization of Rbpms2 fusion proteins in somatic cells of zebrafish embryos. Wild-type GFP-Rbpms2 was detected near the nucleus (~20%) and associated with the centrosomes or spindle (9%), and as in HEK293 cells, GFP-Rbpms2 was localized to granules in 65% of somatic cells of the zebrafish embryo (Figs 3G–3H and 4 1; n = 489 cells, 19 embryos). To determine the identity of these Rbpms2 positive granules, we examined endogenous markers, including the classical stress granule marker Tial-1 and the p-body marker Dcp2, using antibodies previously validated in zebrafish [29]. We detected no overlap with Tial-1 (Fig 3G; n = 172 cells, 8 embryos) and partial overlap with Dcp2 (Fig 3H; n = 317 cells, 11 embryos), indicating that the granules to which GFP-Rbpms2 localizes in zebrafish somatic cells are not stress granules, but that a small subset are GFP-Rbpms2 and Dcp2 positive granules. Therefore, we designate these heterogeneous GFP-Rbpms2 granules as granules of somatic cells. In most GFP-Rbpms2b^ae32^ expressing cells the protein was detected in the nucleus, throughout the cells, and in granules (73%) (Figs 3J and 4; n = 232 cells, 8 embryos). In GFP-Rbpms2a^ae30^ fewer cells with GFP granules were detected (43%). GFP-Rbpms2a^ae30^ was also detected throughout the cytoplasm (33%) or in the nucleus, or in a bipolar pattern likely representing the centrosomes (14% and 10% respectively) (Figs 3K and 4; n = 177, 8 embryos). Strikingly, the sa9329 protein, which disrupts the RNA binding domain was not detected in granules, but strongly localized to the centrosomes/spindle (Figs 3I and 4; n = 311 cells, 9 embryos). These data indicate that the RNA binding and C-terminal domains differentially contribute to Rbpms2 subcellular localization in somatic cells.

**Fig 5:**
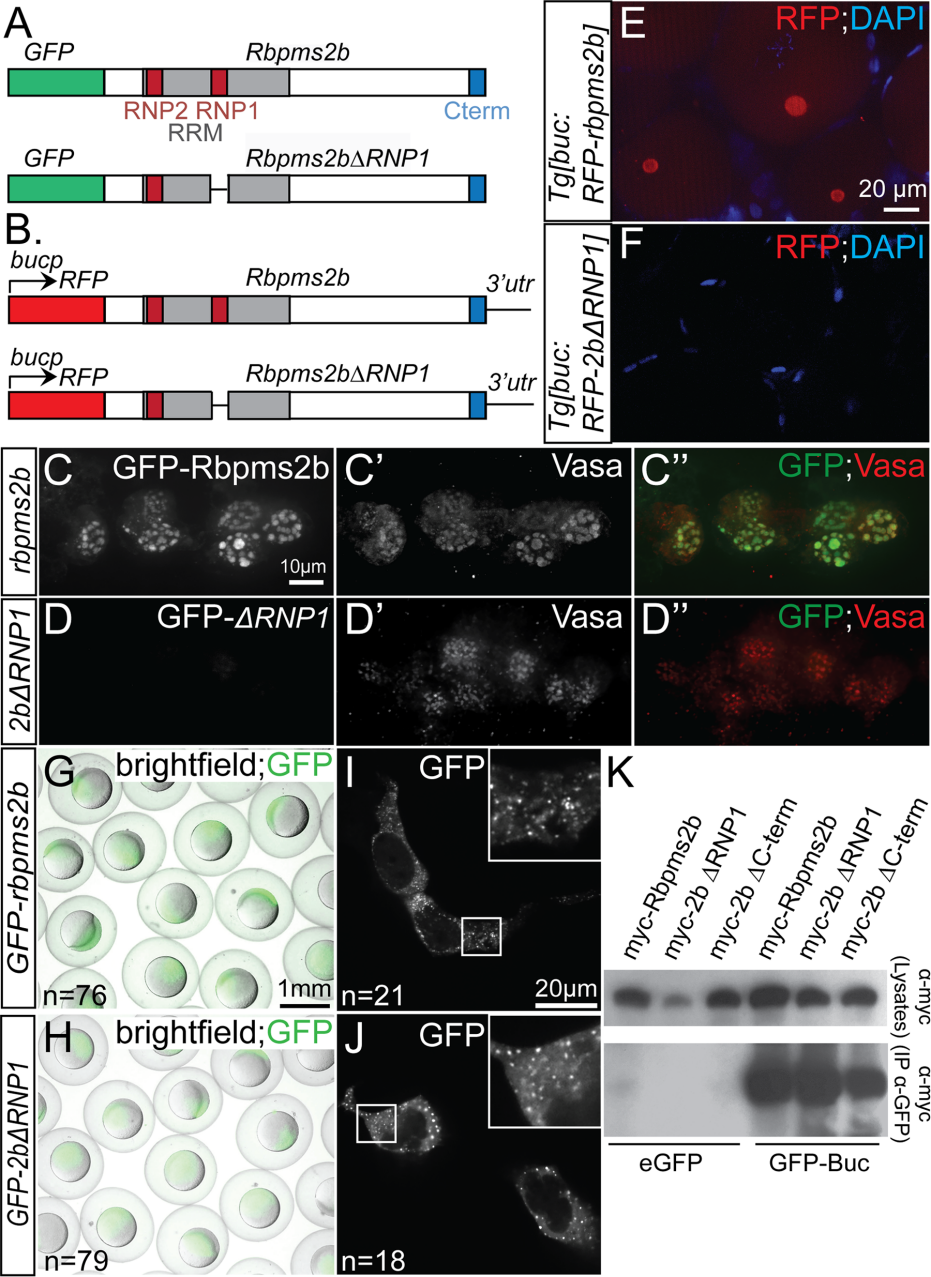
*rbpms2* mutant allele stability and localization activity in germ cells. (A-B) Wild-type GFP-Rbpms2a/b localize to germ granules of PGCs. PGCs are labeled with RFP-*nos3’utr* at 30hpf. (C-E) Like wild-type Rbpms2a and Rbpms2b, GFP-Rbpms2a^ae30^ (C), GFP-Rbpms2b^sa9329^ (D), and GFP-Rbpms2b^ae32^ (E) are all enriched in germ cells. However, GFP-Rbpms2a^ae30^ and GFP-Rbpms2b^sa9329^ are not localized to granules; whereas, GFP-Rbpms2b^ae32^ is partially localized to germ granules. (F) Graph depicting percentage of granule vs. cytoplasmic localization of *GFP-rbpms2* alleles in PGCs. Numbers indicated in parentheses near allele name are number of embryos analyzed, whereas numbers in the right three columns represent numbers of PGCs.

To test if Rbpms2 proteins can localize to granules in the zebrafish germline, we used a commonly employed assay in which *in vitro* transcribed RNA encoding *GFP-rbpms2a/2b* was injected into fertilized zebrafish eggs. For many germ plasm RNAs and interacting proteins, the translated product of the exogenous RNA will localize to germ granules of primordial germ cells (PGCs) in 24-30hpf embryos (reviewed in [1, 30]). PGCs were marked by cytoplasmic expression of RFP-*nanos3’UTR*. Co-injection of RFP-*nanos3’UTR* and either wild-type protein, GFP-Rbpms2a or GFP-Rbpms2b, demonstrated that Rbpms2 fusions localize to germ granules of PGCs. (Fig 5A, 5B and 5F). Next, we checked the germ granule localization capacity of the *rbpms2* mutant alleles: GFP-Rbpms2a^ae30^, GFP-Rbpms2b^ae32^ and GFP-Rbpms2b^sa9329^. Like wild-type Rbpms2 proteins, all mutant proteins became restricted to PGCs (Fig 5C–5E); however, the GFP-Rbpms2a^ae30^ mutant protein and the GFP-Rbpms2b^sa9329^ were cytoplasmic rather than enriched in granules (Fig 5C, 5D and 5F). Similarly, the GFP-Rbpms2b^ae32^ fusion protein was diffuse throughout the cytoplasm in 35% of expressing cells; whereas, in the majority of the cells (65%) GFP-Rbpms2b^ae32^ cells was both diffusely cytoplasmic and enriched in germ granules (Fig 5D and 5F). The PGC-enrichment of the mutant GFP-Rbpms2 alleles suggests that the injected RNAs, or more likely the protein products, are capable of interacting with germ cell factors that stabilize them in germ cells. Because only Rbpms2b^ae32^ retains partial ability to localize to germ granules this suggests that an intact RRM and sequences adjacent to the RRM are required but not sufficient for germ granule localization in PGCs.

### Localization of Rbpms2b to germ granules and the Bb is dependent on an intact RNA-binding domain

**Fig 6:**
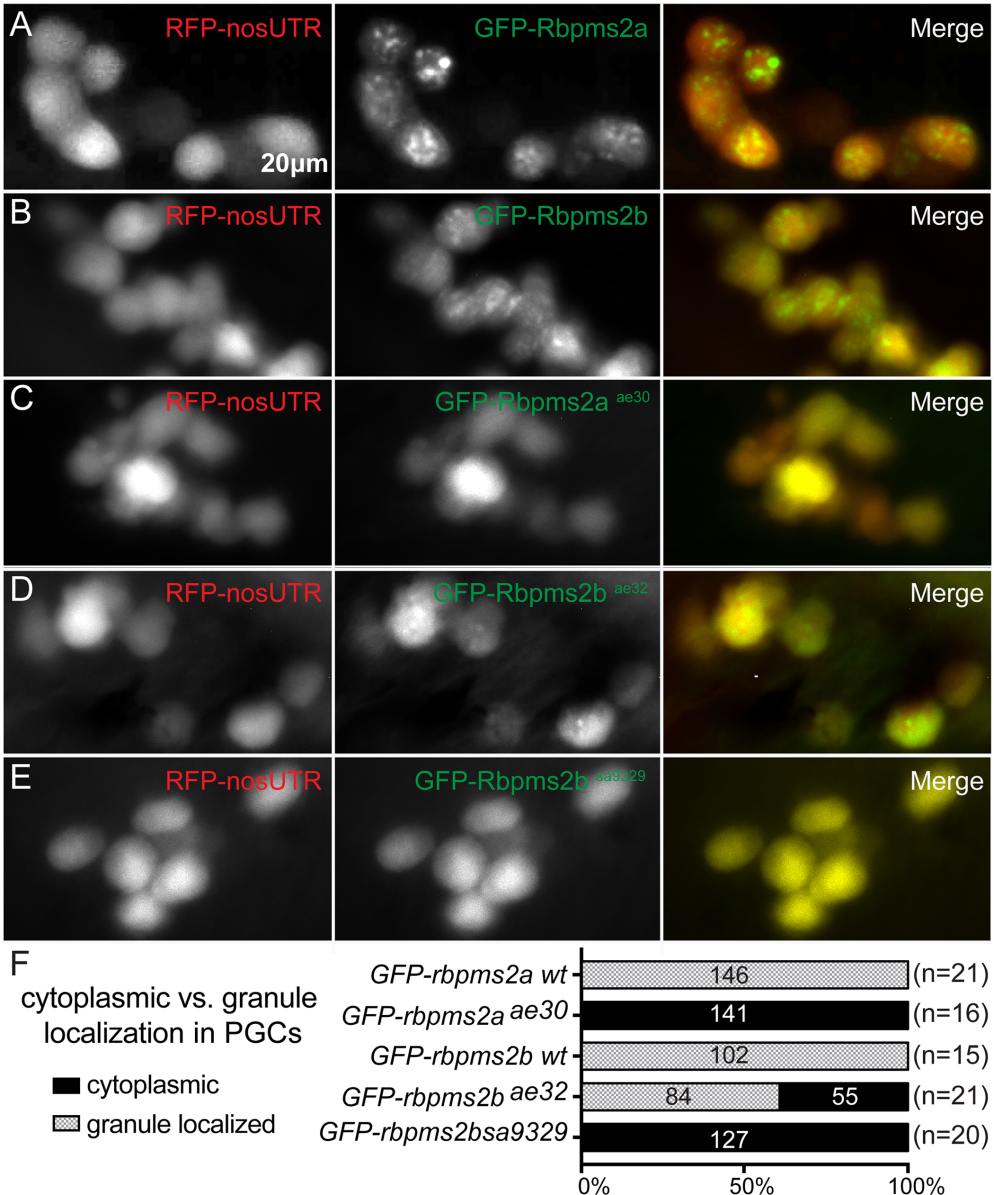
RNA-binding domain is required for Rbpms2 localization in germ cells. (A) Schematic representing constructs used in granule localization assays. (B) Schematic representing transgenic constructs used to express Rbpms2b in oocytes. (C-C”) Wild-type GFP-Rbpms2b localizes with Vasa in germ granules of PGCs. (D-D”) GFP-Rbpms2bΔRNP1, a deletion of 7 residues within the RRM domain, does not localize to germ granules. (E-F) RFP-Rbpms2b expressed in transgenic *Tg[buc*:*RFP-rbpms2b]* ovaries localizes to the Bb (E), while RFP-Rbpms2bΔRNP1 does not (F). (G-J) GFP-Rbpms2b and GFP-Rbpms2bΔRNP1 are stably expressed in gastrula stage embryos (G,H), as well as the granules of HEK293 cells (I, J). (K) GFP immunoprecipitation of Myc- tagged Rbpms2b, Rbpms2bΔRNP1, and Rbpms2bΔC-terminus, a deletion of the last 7 residues within the conserved C-term, demonstrates that the RNP1 domain or conserved C-terminus is not required for interaction with GFP-Bucky ball.

To further investigate the mechanisms that mediate localization to germ and somatic cell granules, we asked if Rbpms2 localization was dependent on its interaction with RNA. To test this, we constructed a mutant version of Rbpms2b, called *GFP-rbpms2bΔRNP1*, that lacks seven amino acid residues of the RNP1 (ribonuclear protein) domain that are essential for making direct contact with the RNA molecule (Fig 6A) [4, 5]. We injected *in vitro* transcribed *GFP-rbpms2b* and *GFP-rbpms2bΔRNP1* to test if the encoded proteins were able to localize to germ granules of PGCs. Wild-type GFP-Rbpms2b was present in germ granules where it localizes with the endogenous germ granule component Vasa; however, GFP-Rbpms2bΔRNP1 did not localize and did not appear to be enriched in PGCs (Fig 6C and 6D”). To determine if failure to localize to germ granules was due to instability of the mutant protein, we examined the injected embryos earlier, at 3-4hpf, and found that both WT and mutant proteins had robust observable GFP signals (Fig 6G and 6H). Furthermore, when these constructs were transfected into HEK293 cells, both wild-type and Rbpms2bΔRNP1 could localize to somatic cell granules (Fig 6I and 6J). Therefore, we determined that GFP-Rbpms2bΔRNP1 produces a stable protein; however, the protein lacking RNP1 cannot localize to the germ granules.

Germ granules in PGCs are similar to the Bb of stage I oocytes, in that both are germline aggregates of RNP particles, and many germ plasm components are localized in both cell-specific structures (reviewed in [1]). To determine fusion protein localization in oocytes, we constructed a transgenic zebrafish line expressing wild-type Rbpms2b under the ovary-specific *buc* promoter [14], *Tg[buc*:*RFP-rbpms2b]*, and a transgenic line that harbors the same RNA-binding deficient mutation described above, *Tg[buc*:*RFP-rbpms2bΔRNP1*] (Fig 6B). When we observed dissected oocytes from adult transgenic females, we found that the wild-type transgenic protein localized to the Bb similar to endogenous Rbpms2. However, as in PGCs, RFP-Rbpms2bΔRNP1 was not localized, and RFP protein was not detectable in oocytes, although transcripts were detectable for both transgenes by *in situ* hybridization (Fig. S4 in S1 Supporting Information and Fig 6E and 6F). The ability of Rbpms2 fusion protein to localize to germ granules or the Bb is likely independent of interaction with Bucky ball, since myc-Rbpms2bΔRNP1, which does not accumulate in germ granules or the Bb can still immunoprecipitate with GFP-Buc in co-IP experiments (Fig 6K). Thus, Rbpms2b requires the RNA-binding RNP1 domain to accumulate in and localize to RNP aggregates of the germline. It remains to be determined if RNP1 contributes to stabilization, efficient translation the protein, or both; however, this domain is dispensable for stability and granule localization in somatic cells such as HEK293. This further suggests that the mechanisms used to localize Rbpms to granules are distinct between germline and soma.

### *rbpms2* mutant adults develop exclusively as males

**Fig 7:**
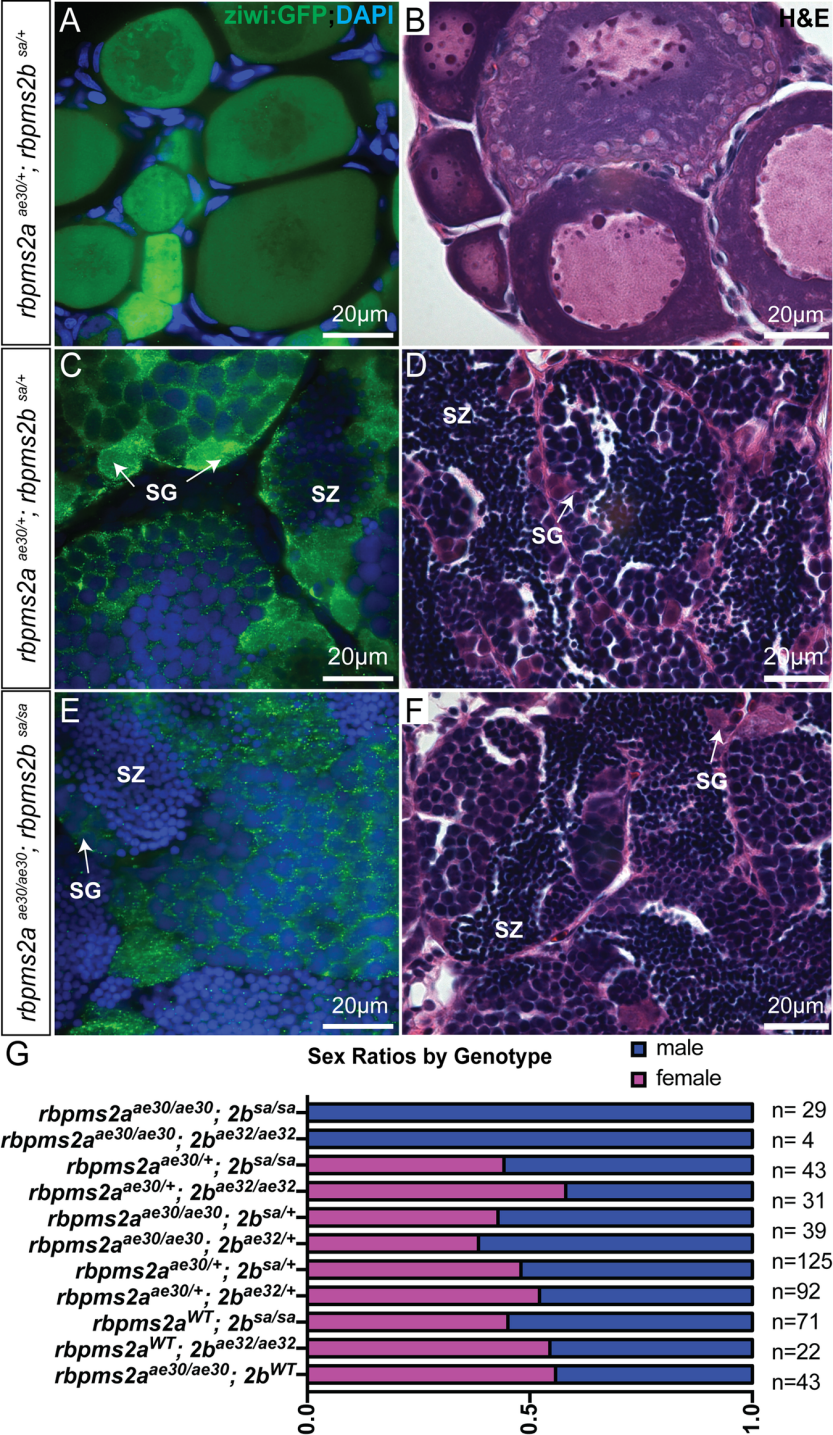
Zebrafish *rbpms2* mutants are fertile males. (A-B) Non-mutant siblings of *rbpms2* double mutants can differentiate and mature as fertile females with overtly normal oocytes observed when germ cells are marked by *ziwi*:*GFP* (A) or with H&E staining (B). (C-D) Non-mutant male siblings of *rbpms2* double mutants display normal spermatogenesis with spermatogonia (SG) and spermatozoa (SZ) detectable with *ziwi*:*GFP* (C) or with H&E staining (D). (E-F) *rbpms2a*^*ae30*^*;rbpms2b*^*sa9329*^ double mutant males have no distinguishable differences in testis morphology or spermatogenic stages from their non-mutant male siblings. (G) Stacked bar chart depicts male to female ratio of adults recovered from each *rbpms2a;rbpms2b* genotype (blue = male, pink = female).

We raised *rbpms2* mutants that had recovered from the edema phenotype in order to assess possible *rbpms2* roles in reproductive development. The adult progeny (>2.5 months) of in-crosses between double heterozygous *rbpms2a*^*ae30/+*^*;rbpms2b*^*sa9329/+*^ fish were genotyped, and *rbpms2a* or *rbpms2b* single mutants and double heterozygotes or heterozygote-mutants displayed typical 50/50 male-female sex ratios (Fig 7A–7D and 7G). However, no *rbpms2* DM adults were identified among 129 fish, although approximately 8 would be expected according to Mendelian genetics (at a prevalence of 1:16). To overcome a potential survival disadvantage of *rbpms2* mutants, we sorted mutants based on their transient edema phenotype at d3 to determine if *rbpms2* mutant adults could be recovered when reared separately from their wild-type siblings. Using this strategy, we recovered 33 *rbpms2* mutants to adulthood, all of which were fertile males (Fig 7E–7G). Fertility of *rbpms2* mutant males was indistinguishable from wild-type males based on mating assays (fertilization of eggs). Additionally, histological evaluation of mutant testes, through H&E staining and expression of a transgene *[ziwi*:*GFP]* that marks germ cells of both sexes [31], revealed no differences from normal male siblings (Fig 7C–7F). Undifferentiated spermatogonia were noted on the basement membrane of the testicular tubules, as well as differentiated spermatozoa within tubule lumens, indicating that progression of spermatogenesis is normal in *rbpms2* mutants.

### *rbpms2* mutants undergo normal germline development until sexual maturation

**Fig 8:**
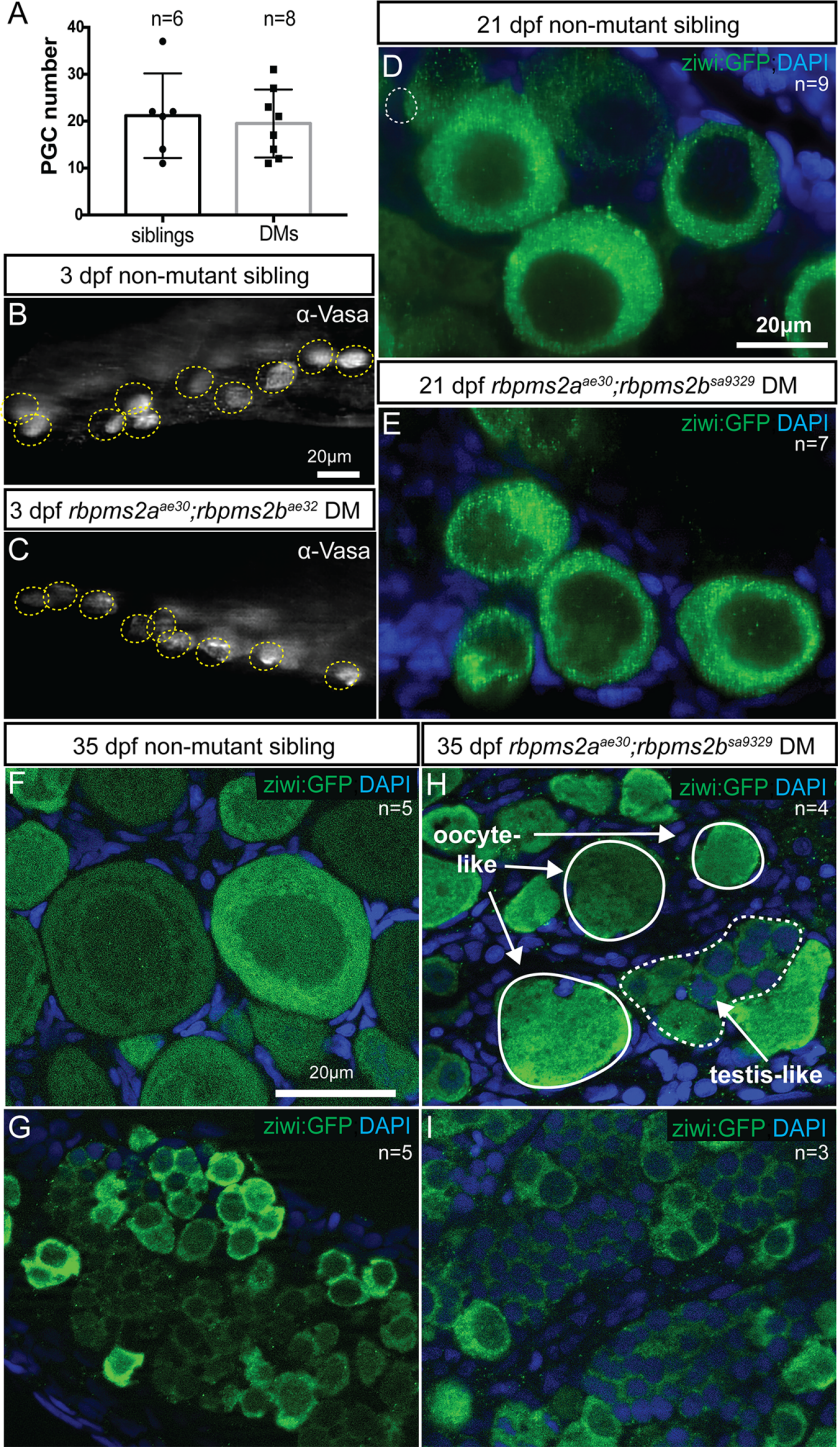
*rbpms2* is required for definitive female differentiation. (A) Quantification of PGC numbers in d3 *rbpms2* double mutants and non-mutant siblings. (B-C) Vasa staining to count PGCs in a representative slice from Z-series with PGCs circled in yellow in non-mutant sibling (B), and *rbpms2* double mutant (C). (D-E) Transgenic germ cell marker *ziwi*:*GFP* allows visualization of comparable gonocyte development in bipotential gonads of d21 non-mutant siblings (D) and *rbpms2* mutants (E). (F-G) Female (F) and male (G) gonad differentiation of control non-mutant siblings at d35. (H) *rbpms2* mutant gonad with oocyte-like cells outlined in solid line, and testis-like cells outlined in dashed line. (I) *rbpms2* mutant gonad resembling non-mutant male siblings.

Zebrafish will differentiate as males if germ cell numbers are diminished or oogenesis fails. Early in embryonic development, germline differentiation to the male fate can result when there are few or no PGCs [32–35]. To test the possibility that the all-male phenotype of *rbpms2* mutants was caused by insufficient PGC numbers, we stained d3 *rbpms2* mutants and their siblings for the germ cell marker Vasa [36]. PGCs were examined by confocal imaging of the lateral side followed by manual counting through the Z series. We found no significant difference in PGC numbers between non-mutant siblings and *rbpms2* mutant embryos, with both groups having approximately 20 PGCs per side (Fig 8A–8C). Next, we examined d21 bipotential gonads in the *[ziwi*:*GFP]* transgenic line and determined that development of primitive oocytes/gonocytes and meiotic initiation were comparable between *rbpms2* mutants and their non-mutant siblings (Fig 8D and 8E).

**Fig 9:**
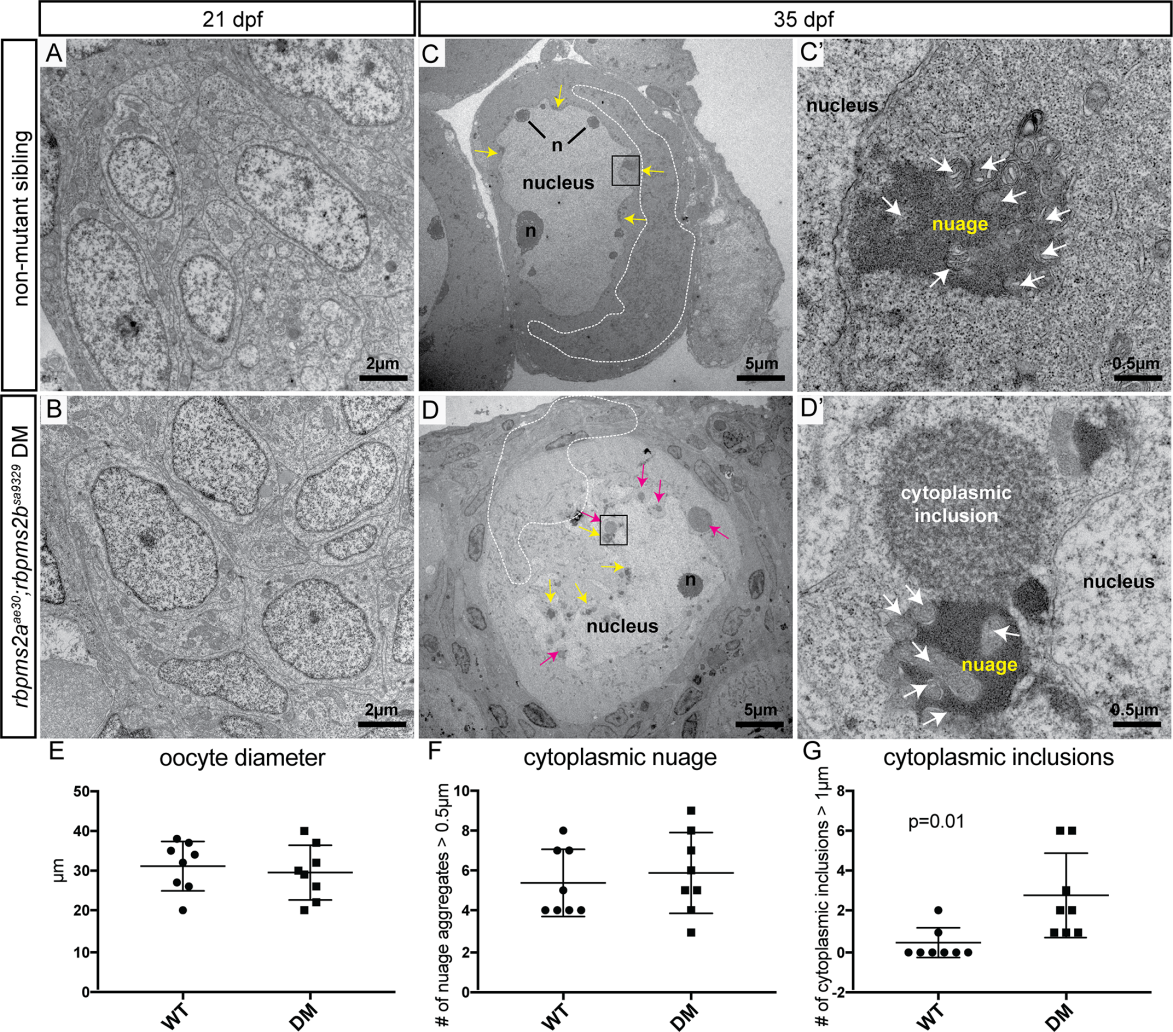
*rbpms2* mutant oocytes contain atypical cytoplasmic inclusions. (A-B) Comparable clusters of gonocytes from d21 non-mutant siblings (A), and *rbpms2* double mutants (B). (C,D) Oocytes from d35 non-mutant siblings (C), and *rbpms2* double mutants (D). Marked features include: n = nucleoli, white dotted line outlining areas of mitochondrial enrichment, yellow arrows indicating nuage accumulations, pink arrows indicating atypical cytoplasmic inclusions. Areas marked in a black box in (C, D) are magnified in (C’,D’). (C’, D’) High magnification images of nuage with associated mitochondria (white arrows) in non-mutant oocyte (C’), and juxtaposed nuage and atypical cytoplasmic inclusion in *rbpms2* mutant oocyte (D’). (E-G) Oocytes of comparable diameter (E) were used to quantify and compare number of nuage aggregates over 0.5μm (F), and number of cytoplasmic inclusions figover 1μm (G). Significance was analyzed with unpaired t-test.

Next, we examined *rbpms2* mutants and siblings at d35 when sexual differentiation of the gonads has been initiated. In non-mutant siblings, we found readily distinguishable ovary tissue indicative of female differentiation with numerous stage I and II oocytes (Fig 8F), or testis differentiation with a few gonocytes but mostly small spermatogonia-like cells (Fig 8G). In contrast, *rbpms2* mutant gonads were either wholly testis-like (resembling gonads of their non-mutant male siblings at d35) (Fig 8I), or were of mixed character with some spermatogonia-like cells, and also significant numbers of oocyte-like germ cells based on cell diameter and nuclear morphology (Fig 8H). We quantified the size of *[ziwi*:*GFP]*-labeled oocytes at d35 and found that non-mutant oocytes reached average diameters of up to 57±5 μm (n = 26 oocytes, 3 fish); whereas, the largest *rbpms2* mutant oocytes reached average diameters of up to 31±4 μm (n = 23 oocytes, 4 fish). These diameters are consistent with *rbpms2* mutants failing in oogenesis sometime during the growth phase of stage Ib oocytes (which typically have diameters of 20–140 μm) [37]. Consistent with this notion, ultrastructural examination of d21 gonads by transmission electron microscopy revealed no discernable differences in nuclear or cytoplasmic morphology of gonocytes (n = 3 *rbpms2* mutants, n = 4 wild-type) (Fig 9A and 9B). Taken together, these results suggest that intersex *rbpms2* mutants initiate oogenesis, but are in the process of transitioning to the male phenotype, since ultimately all recovered *rbpms2* mutants are fertile males.

### Oocyte loss and male development of *rbpms2* mutants are *p53-*independent

In zebrafish the transition to male fate has been reported to involve apoptosis of germ cells [38]. Consistent with this notion, disrupting the key regulator of apoptosis, Tp53, can suppress loss of the female germline and the eventual male-only phenotypes of zebrafish mutants disrupting *zar1*, *brca2* or *fancl* [39–41]. These studies indicate that apoptosis of ovary-like cells of the bipotential gonad facilitates the transition to testis. Therefore, we reasoned that suppressing *tp53-*mediated apoptosis by genetically eliminating *tp53* may support sustained development of oocytes in *rbpms2* mutants. To investigate whether loss of *tp53* could support oocyte development, we generated triple mutants with *rbpms2* and *tp53*^*M214K*^ mutations (Berghmans et al., 2005) and examined the gonads of *rbpms2a*^*ae30/ae30*^*;rbpms2* ^*ae32/ae32*^*;tp53*^*M214K/M214K*^ fish at d35, when gonad differentiation into testis or ovary has occurred. At this stage, we detected no differences between the gonads of *rbpms2* mutants and *rbpms2* mutants lacking Tp53 (n = 5) (Fig. S5 in S1 Supporting Information), and more advanced stages of oogenesis were not recovered. Moreover, *rbpms2;tp53* triple mutants developed exclusively as fertile adult males, like *rbpms2* double mutants (n = 3). Based on this analysis, we conclude that loss of *tp53* is not sufficient to support further ovary differentiation in *rbpms2* mutants. Moreover, differentiation of *rbpms2* mutants as males occurs by a mechanism that is independent of the p53-mediated apoptotic pathway.

### Aberrant cytoplasmic inclusions and Bucky ball structures in *rbpms2* mutants

To examine the subcellular compartment of *rbpms2* mutant oocytes more closely, we compared the ultrastructure of d35 gonads from wild-type females (n = 2) and *rbpms2* mutants containing oocyte-like germ cells (n = 2). We found both wild-type and *rbpms2* mutant oocytes had accumulated asymmetric mitochondria on one side of the nucleus, a hallmark of early oocyte polarization and Bb formation (Fig 9C and 9D, mitochondrial accumulation outlined in white) [15, 42]. In general, oocytes in the wild-type gonads tended to be larger; therefore, we focused our analysis on oocytes with similar diameters in both genotypes (ranging from 20–40 μm) (Fig 9E). We also examined these oocytes for the presence of characteristic nuage: discrete areas of cytoplasm that appear as electron-dense, fibrous accumulations, closely associated with the nuclear envelope and mitochondria [43]. We found similar numbers of cytoplasmic nuage accumulations in wild-type and *rbpms2* mutant oocytes (Fig 9C, 9D and 9F, yellow arrows). However, *rbpms2* mutant oocytes also contained atypical cytoplasmic inclusions with electron density distinct from nuage that excluded mitochondria (Fig 9D, 9D’ and 9G); atypical cytoplasmic inclusions >1μm were rarely observed in wild-type oocytes (p = 0.01, unpaired t-test). In *rbpms2* mutants, cytoplasmic inclusions were often significantly large, with three of the eight analyzed oocytes containing structures measuring over 3μm across. Although these large electron dense structures are a feature of *rbpms2* mutant oocytes, the molecular contents of these cytoplasmic bodies remain to be determined.

**Fig 10:**
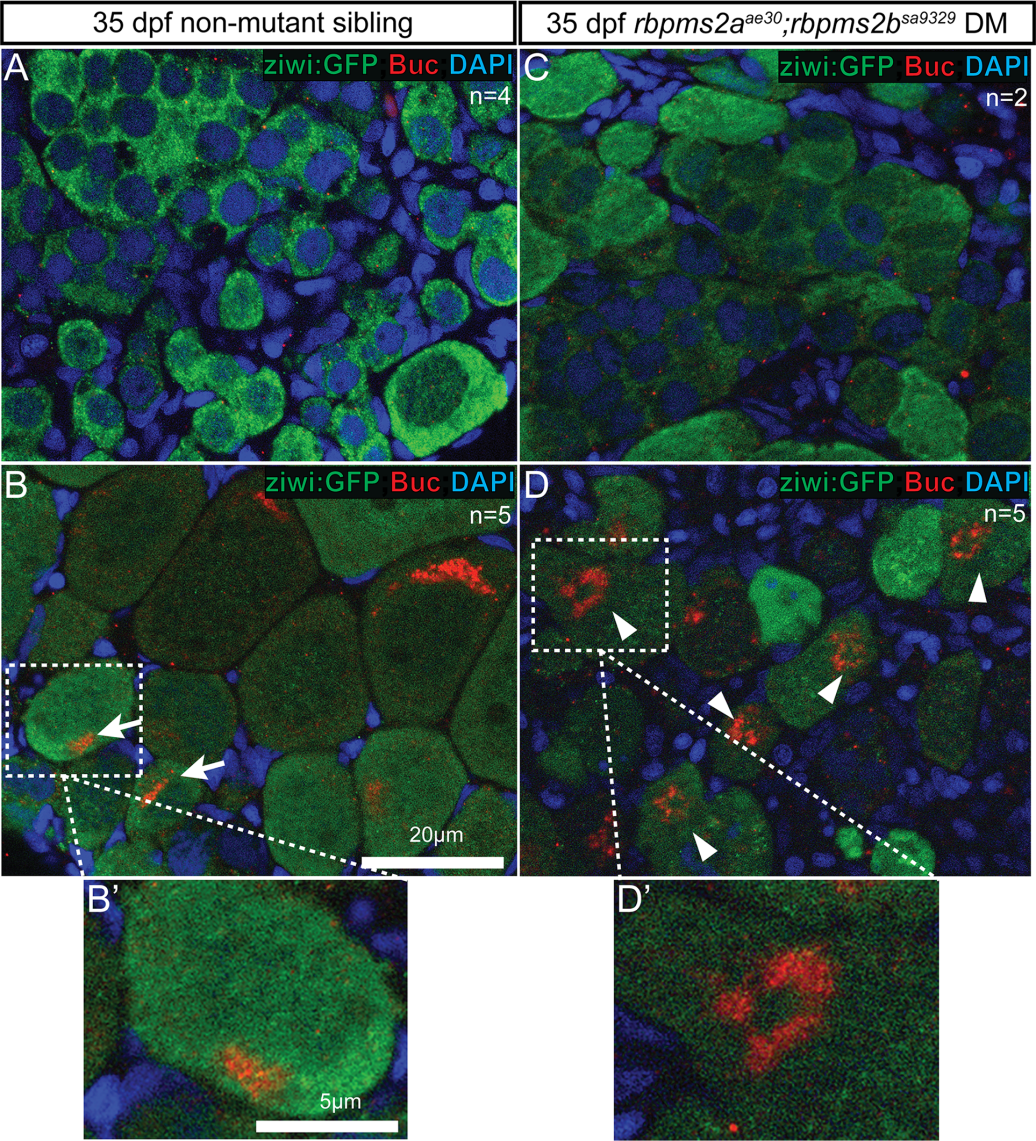
Bucky ball stained structures are abnormal in *rbpms2* mutants. (A) Non-mutant sibling males have no specific Buc signal. (B) Non-mutant sibling female with asymmetric Buc localization in compact perinuclear domain of primary (stage I) oocytes (arrows). (B’) Magnified view of normal Buc localization in non-mutant sibling oocyte. (C) Like their control male siblings, differentiated male *rbpms2* mutants have no Buc signal. (D) Oocyte-like cells of *rbpms2* mutant gonads have more dispersed asymmetric Buc staining, frequently observed in a ring-like pattern (arrow-heads). (D’) Magnified view of ring-like Buc localization in *rbpms2* mutant oocyte.

We have previously shown that Rbpms2 C-terminus interacts with Bucky ball protein and *buc* transcripts (Fig. S6A in S1 Supporting Information) [14]; therefore, we assessed whether loss of Rbpms2 affects Bucky ball abundance or localization by examining Bucky ball protein in Rbpms2 mutants. As previously reported, no specific Buc staining was apparent in d35 testes, including in larger gonocytes of males (Fig 10A), and Buc protein was expressed in pre-Bb stage oocytes at d35 (Fig 10B) [14]. Accordingly, no Buc staining was observed in d35 *rbpms2* mutant gonads that had already undergone testis differentiation (Fig 10C). Consistent with the notion that a subset of *rbpms2* mutants initiated oogenesis as judged by this marker, we detected asymmetric Buc staining in germ cells of *rbpms2* mutants that retained oocyte-like cells. As previously reported, the morphology of the Buc stained structures in non-mutant primary oocytes was compact and perinuclear (Fig 10B and 10B’ white arrows). Interestingly, *rbpms2* mutant oocytes had more dispersed Buc distribution that formed a ring-like structure (Fig 10D and 10D’ white arrow heads). Although perinuclear localization of Vasa was detected, Vasa was also more dispersed in *rbpms2* mutant oocytes (Fig. S3 in S1 Supporting Information). Whereas another Balbiani body localized protein, Macf1/Mgn [44] was not detected in these early stage oocytes (Fig. S3 in S1 Supporting Information). Therefore, Macf1 protein may only accumulate in more mature Balbiani bodies, consistent with its role in Balbiani body dispersal [44, 45]. Thus, we conclude that Rbpms2 is not required for initial Buc or Vasa translation, but may play a role in localizing Buc protein or regulating Bb morphology.

## Discussion

Our lab has previously shown that zebrafish Rbpms2b interacts with the Bucky ball protein (Heim et al., 2014). This protein interaction is conserved in *Xenopus*, where Velo1 (Buc homolog) can interact with Hermes (Rbpms family) (Nijjar and Woodland, 2013). However, *Xenopus* has a single reported *rbpms* gene, *hermes*, while zebrafish have three *rbpms* genes: *rbpms (rbpms1)*, *rbpms2a* and *rbpms2b*. We used phylogenetic analysis based on protein sequence similarity to show that the duplicated zebrafish *rbpms2* genes are most closely related to *hermes*. This is in agreement with the conserved embryonic expression patterns of zebrafish *rbpms2a* and *rbpms2b* in the embryonic heart primordium, pronephros and retina; a tissue-specific expression pattern similar to those reported for *hermes* homologs in *Xenopus*, chicken and mouse (Gerber et al., 2002). In contrast, zebrafish *rbpms* expression is weak and not tissue-restricted at 24 and 48 hpf, indicating that this gene may be functionally divergent from *rbpms2*.

The *hermes* RNA and protein are localized to the Balbiani body in *Xenopus* primary oocytes (Zearfoss et al., 2004). We showed that all three zebrafish *rbpms* transcripts are expressed in primary oocytes, however only *rbpms2a* is enriched in the Bb. Previously, studies using a cross-reacting anti-Hermes antibody on zebrafish oocytes have shown that a homolog of Hermes also localizes to the Bb in zebrafish (Kosaka et al., 2007; Marlow and Mullins, 2008). Accordingly, using two commercial antibodies raised against human Rbpms2, we determined that zebrafish Rbpms2 homologs are enriched in the Bb. While we also tested several commercially available Rbpms (Rbpms1) antibodies in the zebrafish ovary, it could not be determined if these antibodies were simply not cross-reactive, or if Rbpms protein is not localized to the Bb, and thereby is indistinguishable above background. Both Rbpms2a and Rbpms2b are likely expressed and enriched within the Bb, because the *rbpms2b*^*ae32/ae32*^ mutant ovary showed Bb- enriched Rbpms2 (Rbpms2a), and the converse was also true for the *rbpms2a*^*a30/ae30*^ mutant ovary (enriched Rbpms2b). We conclude that Rbpms2a and Rbpms2b are expressed and enriched in the Bb of primary oocytes.

### Distinct mechanisms of Rbpms2 localization to granules in somatic and germ cells

Stress granules are ribonucleoprotein particles formed in the cytoplasm of somatic cells during stress responses that stall the initiation of protein translation; for example, stress granules form in response to environmental stress such as heat shock, translation-initiation blocking drugs, or in response to overexpression of RNA-binding proteins that inhibit translation (reviewed in (Buchan and Parker, 2009; Panas et al., 2016)). Stress granules store mRNAs that are in the process of translation initiation, and typically contain numerous components of translation initiation complexes including poly(A) positive mRNAs, the 40s ribosomal subunit, numerous eukaryotic initiation factor proteins (eIF2, 3 and 4), as well as RNA helicases, and other RNAbps (reviewed in (Buchan and Parker, 2009; Kedersha and Anderson, 2009). Previous work on human RBPMS and RBPMS2 has found that these proteins partially overlap with poly(A)+ mRNA and the stress granule marker G3BP1 (GTPAse SH3-Domain Binding Protein/ Stress Granule Assembly factor 1), consistent with a possible role in translational repression in the stress granule (Farazi et al., 2014).

**Fig 11:**
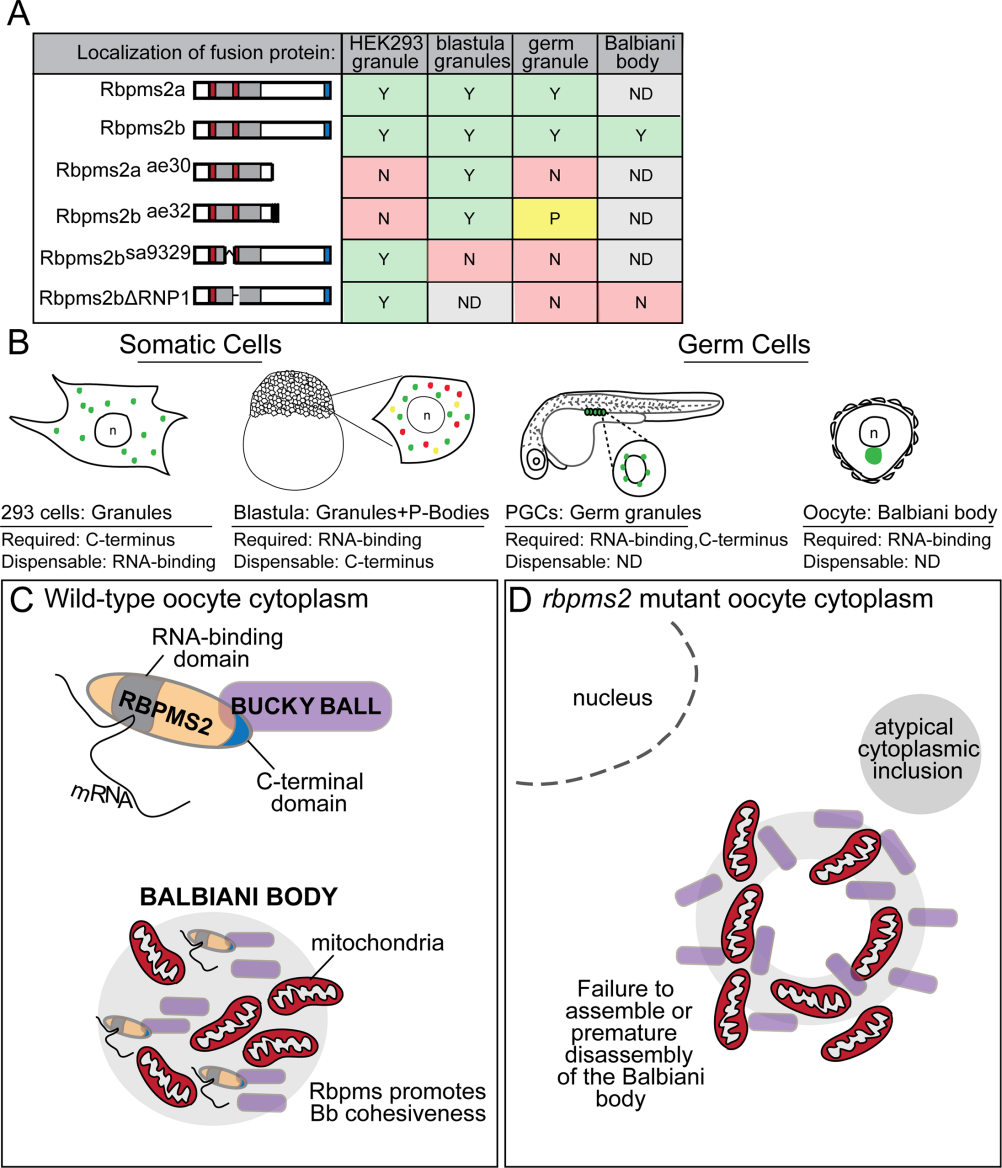
Summary and model depicting Rbpms2 localizing domains, and consequences of *rbpms2* loss on Buc and Balbiani body architecture. (A) Chart depicting wild-type and mutant Rbpms2 proteins and the contribution of Rbpms2 functional domains to protein localization within subcellular aggregates of somatic and germline cell types. Y = yes, N = no, P = partial, ND = not determined. (B) In 293 (somatic) cells Rbpms2 (green) requires its C-terminus, but not the RNA binding domain for somatic cell granule localization; whereas, in somatic cells of the zebrafish blastula Rbpms2 accumulates in P-bodies (yellow and red dots) and Dcp2 negative granules (green dots) but not Tial-1 positive stress granules and requires an intact RNA binding domain. Similarly, in germline cells (PGCs and oocytes) the RNA binding domain, and presumably RNA binding is required for localization to germ granules and the Balbiani body, and the C-terminus contributes to the wild-type localization of the proteins. (C) Rbpms2 in wild-type primary oocytes promotes Balbiani body assembly or cohesiveness possibly through interaction with Bucky ball protein via the Rbpms2 C-terminus or via interaction with *buc* RNA. (D) In *rbpms2* double mutants, mitochondria are asymmetrically present within early oocytes and associate with nuage. Although Bucky ball protein is translated, no cohesive Buc structure forms in the Bb. Instead the Bucky ball domain is scattered and donut shaped, and large atypical electron dense bodies form in the cytoplasm, reflecting either failure to assemble the Balbiani body or its premature disassembly.

We found that zebrafish fusion proteins containing wild-type GFP-Rbpm2a or GFP-Rbpms2b localize to punctate structures in the cytoplasm of HEK 293 and zebrafish somatic cells (Fig 10A and 10B). In zebrafish somatic cells, these granules partially overlapped with the p-body marker Dcp2 but not the stress granule marker Tial-1. Granule localization was disrupted for GFP-Rbpms2a^ae30^ and GFP-Rbpms2b^ae32^, which are expressed in the cytoplasm of HEK 293 and in the cytoplasm and nucleus of zebrafish blastomeres. In HEK 293 cells, localization was only partially impaired in GFP-Rbpms2b^sa9329^, which was not in granules but instead showed a striking localization to the centrosome in zebrafish somatic cells (Fig 11A and 11B). Interestingly, the C-terminal part of Rbpms2 that is missing from GFP-Rbpms2a^ae30^ and GFP-Rbpms2b^ae32^ (which have intact residues 1–107 and 1–116, respectively) has previously been shown to be dispensable for RNA-binding in electrophoretic mobility shift assays (EMSAs) (Farazi et al., 2014), and for dimerization (Sagnol et al., 2014). Furthermore, GFP-Rbpms2b^sa9329^, which partially lacks RNA-binding residues coded for by the (skipped) third exon, and GFP-Rbpms2bΔRNP1, which lacks all the RNA-binding residues of RNP1, can still localize to granules in HEK 293 cells and to the centrosome of zebrafish somatic cells, where the wild-type protein can also be detected (Fig 10A and 10B). Thus, the abnormal localization of the truncated mutant proteins GFP-Rbpms2a^ae30^ and GFP-Rbpms2b^ae32^, is likely not due to inability to bind RNA or dimerize. This suggests that perhaps another protein interaction that is mediated through the C-terminus of RBPMS2 facilitates recruitment of these proteins to somatic cell granules.

The germ granules of PGCs share many components and general features of somatic cell stress granules and p-bodies, but also contain RNAs and proteins that are unique to the germline (reviewed in (Voronina et al., 2011)). Injection of exogenous GFP-tagged *rbpms2a/b* RNAs resulted in the localization of the translated proteins to the PGC germ granules. Localization of all three mutant proteins, GFP-Rbpms2a^ae30^, GFP-Rbpms2b^ae32^, and GFP-Rbpms2b^sa9329^, was absent or severely reduced in germ granules although diffuse expression in the germ cell cytoplasm was robust (Fig 11A and 11B). However, unlike in somatic cell granules, the lack of the RNP1 motif in GFP-Rbpms2bΔRNP1 prevented this mutant fusion protein from becoming enriched in PGCs (Fig 10A and 10B). Based on this result, we speculate that interaction with RNA is likely required for enrichment or stabilization of Rbpms2 proteins to the PGC cytoplasmic compartment; however, localization within the granules depends on a separate mechanism. A similar phenomenon may operate for the localization and stability of Rbpms2 in the oocyte Balbiani body because the fusion protein coded for by the transgenic line *[buc*:*RFP-rbpms2b]* localizes to the Bb like the endogenous protein; however, the product of *[buc*:*RFP-rbpms2bΔRNP1*] transgenics does not localize to the Bb or yield detectable stable protein, despite comparable transcript expression from both transgenes (Fig 11A and 11B). Therefore, Rbpms2 interaction with germ plasm RNAs may be required for efficient translation of *rbpms2* RNA or stability of the protein in germ cells including primordial germ cells and the primary oocyte.

### Rbpms2 functional redundancy

We predicted that the zebrafish *rbpms2a* and *rbpms2b*, might function redundantly in the germline based on their overlapping expression domains, and their extremely similar protein sequences. Furthermore, Rbpms2a and Rbpms2b can both interact with Bucky ball protein in co-transfection and co-IP experiments. Thus, we strategically targeted both *rbpms2* genes for Crispr-Cas9 mutagenesis. Indeed, single mutants for *rbpms2a* or *rbpms2b* had no discernable embryonic or adult phenotypes, supporting our prediction of functional redundancy between these genes. However, loss of both *rbpm2a* and *rbpms2b* resulted in cardiac phenotypes in zebrafish embryos, consistent with the reported role for *hermes* in embryonic heart development (Gerber et al., 2002; Gerber et al., 1999). Furthermore, loss of both *rbpms2* genes resulted in defective oocyte differentiation, and a male only phenotype, confirming that these genes are likely redundant in their reproductive functions.

### Rbpms2 is required for oocyte differentiation

Of the adult *rbpms2a;rbpms2b* double mutants (*rbpms2* mutants) that escaped the embryonic cardiac phenotype, we could recover only fertile males. Due to the complex manner in which zebrafish sexual differentiation is regulated, we reasoned that this all-male phenotype was likely the result of a defect in an *rbpms2*-dependent aspect of germline development. Nonetheless, we observed comparable gonad development between *rbpms2* mutants and their non-mutant siblings during early embryonic development (d3) when mutants and siblings had similar numbers of PGCs, and later at the bipotential stage (d21) when mutants and siblings had comparable germ cell morphologies and composition of meiotic-stage gonocytes.

At the onset of sexual differentiation (d35), approximately half of the wild-type siblings will differentiate ovaries containing numerous primary oocytes, and the other half develop primitive testes containing numerous spermatogonia that are characteristically small in size, with high nuclear-to-cytoplasmic ratios, and present in large clusters. However, gonads of *rbpms2* mutants do not complete differentiation as ovaries and instead were categorized as either testis-like gonads resembling those of their male siblings, or intersex gonads with some spermatogonia, but also many germ cells that morphologically resemble oocytes. Many of the *rbpms2* mutant oocytes were significantly large, reaching diameters comparable to the oocytes of their siblings. Furthermore, *rbpms2* mutants express the female-specific marker Bucky ball in their oocytes (Bontems et al., 2009; Heim et al., 2014). Importantly, it should be noted that the role of *rbpms2* in promoting oogenesis is Bucky ball-independent, since zygotic *buc* mutants progress normally through oogenesis, and can become adult females with mature ovaries (although *buc* mutant eggs are un-patterned) (Bontems et al., 2009; Dosch et al., 2004; Heim et al., 2014; Marlow and Mullins, 2008). In contrast, *rbpms2* mutants initiate oocyte differentiation and maturation; however, mutant oocytes ultimately fail to complete differentiation and are lost in a *tp53-*independent manner, resulting in apparently normal spermatogenesis.

### Rbpms2 roles in oocyte polarity and Bb architecture

Rbpms2 protein interacts with the Bucky ball protein and RNA transcript (Heim et al., 2014); therefore, we examined Bucky ball protein in *rbpms2* mutant oocytes to determine if Bucky ball abundance or localization is Rbpms2-dependent. The presence and asymmetric pattern of Buc localization in *rbpms2* mutant oocytes indicates that Rbpms2 is not required for translation of the *bucky ball* transcript. This is in agreement with other studies suggesting that *Xenopus* Hermes and human RBPMS proteins are likely to be translational repressors, rather than activators (Farazi et al., 2014; Song et al., 2007); however, it is also possible that other RNA-binding proteins, like the related Rbpms(1), act redundantly with Rbpms2 to promote *buc* translation. In wild-type oocytes prior to the Bb stage, Buc staining typically appears as a crescent abutting the oocyte nucleus and then coalesces to form a spherical Balbiani body (Heim et al., 2014) (Fig 11C). However, in *rbpms2* mutant oocytes we observed more diffuse Buc staining that formed a ring-like structure not observed in wild-type oocytes. Therefore, Rbpms2 may play a role in localizing Buc protein through its C-terminus, possibly by bringing Buc molecules into proximity to promote their oligomerization, or regulating Bb morphology by interactions with other Bb localized proteins, or by preventing translation of a protein that promotes Bb disassembly (Fig 11C and 11D). The only protein known to promote Bb disassembly in zebrafish is Mgn/Macf1 [44, 45]; however, we did not detect Mgn/Macf1 in the early stage oocytes that are present in *rbpms2* mutants, although Vasa and Buc were detected. Thus, it seems unlikely that premature activation of Macf1/Mgn accounts for the dispersed Buc and Vasa in *rbpms2* mutants. Examination of ultrastructure with TEM in primary oocytes determined that asymmetric mitochondrial localization or accumulation of nuage is not dependent on *rbpms2*; however, the presence of atypical cytoplasmic inclusions is significantly enhanced in the mutant population. It seems plausible that these electron dense structures might interfere with proper Bb coalescence; thus, Rbpms2 would limit a factor that is disruptive to Bb assembly. Identification and further characterization of additional Bb-localized components is needed to determine if this peculiar localization pattern is specific to Bucky ball protein, or reflects a pattern of exclusion of Bb components such as mitochondria and germ plasm by the large electron dense structures present in *rbpms2* mutants.

In summary, using transient and transgenic localization assays we identified unique domains of Rbpms2 that mediate its localization to subcellular structures of germ cells and somatic cells. We determined that an intact RNA binding domain is dispensable for Rbpms2 localization to granules of HEK 293 cells, and Rbpms2 subcellular localization to the centrosome in somatic cells of the zebrafish blastula, but is required for Rbpms2 association with germ granules in primordial germ cells and the Balbiani body of oocytes. Using CRISPR-Cas9 mutagenesis we showed that the two zebrafish Rbpms2 genes function redundantly in heart development and in oogenesis. Establishment of oocyte polarity and initial translation of Buc and Vasa proteins do not require Rbpms2 protein; however, Rbpms2 is required for normal Balbiani body structure and to prevent formation of aberrant cytoplasmic bodies. In addition to its role in preserving Balbiani body architecture, we discovered an independent and novel role for Rbpms2 in maintaining ovary fate. Further analyses are required to determine how Rbpms2 fits into the current framework of factors known to promote oogenesis.

## Materials and methods

### Zebrafish

Wild-type zebrafish embryos of the AB strain were obtained from pairwise matings and reared according to standard procedures [46]. Embryos were raised in 1X Embryo Medium at 28.5°C and staged as described [47]. All procedures and experimental protocols were in accordance with NIH guidelines and approved by the Einstein (protocol #20140502) and ISMMS (protocol # 17–0758 INIT) IACUCs. The zebrafish *rbpms2b*^*sa9329*^ allele was obtained from the Sanger Institute’s Zebrafish Mutation Project [24]. The zebrafish transgenic reporter lines *ziwi*:*GFP* and *cyp19a1a*:*GFP* were obtained from Bruce W. Draper [22, 31].

### Mutagenesis

Zebrafish *rbpms2a*^*ae30*^ *and rbpms2b*^*ae32*^ mutants were made using CRISPR-Cas9-mediated mutagenesis as described [23]. Guide RNAs (*gRNA*) targeting the fifth exon of *rbpms2a* and *rbpms2b* were designed using the open-access ZiFiT Targeter website (https://crispr-cas9.com/96/zifit-targeter-crispr-cas9/), resulting in the targeting sequences of 5’-GGGTGCAGGTTGGAAGGGTT-3’ for *rbpms2a* and 5’-GGGTGGATATTTGTGGGATT-3’ for *rbpms2b*. The *rbpms2* targeting oligos were ligated into the gRNA expression vector pDR274 (Addgene Plasmid #42250). The *gRNAs* were synthesized using MAXIscript T7 Kit (Life Technologies, AM1312M). The Cas9 RNA (Addgene Plasmid #42251) was synthesized using mMESSAGE MACHINE SP6 Transcription Kit (Life Technologies, AM1340) and Poly(A) Tailing Kit (Life Technologies, AM1350). 100ng of *gRNA* was co-injected with 300ng of *cas9* RNA into the cytoplasm of 1-celled zebrafish embryos. T7 Endonuclease I assays (NEB, M0302) and sequencing were used to confirm mutagenesis at d2-3. Injected embryos were raised to adulthood and their progeny were screened to identify founders with germline mutations. Identified alleles were outcrossed to wild-type AB fish prior to incrossing.

### Genotyping

Genomic DNA was extracted from adult fins or single embryos using standard procedures [46]. The genomic region surrounding *rbpms2a*^*ae30*^ was amplified using the primers 5’-GGGAAGCACCGCTTACAATA-3’ and 5’- TTTGACTCACATGGGTCTCG-3’, followed by digestion of the wild-type strand with the enzyme BsurI (ThermoFisher, FD0154). The genomic region surrounding *rbpms2b*^*ae32*^ was amplified using the primers 5’- GCGTGTAGTTTGTGTCCACC-3’ and 5’- TGTGGGCCGGAAACTTACAT-3’, followed by digestion of the mutant strand with the enzyme EcoRV (ThermoFisher, FD0304). Finally, the genomic region surrounding *rbpms2b*^*sa9329*^ was amplified using the dCAPs primers 5’- CACTTATCAAGCTAACTTCAAAGCAGC-3’ and 5’- TGAAAGGGGACAAATAAGTCA-3’, followed by digestion of the wild-type strand with the enzyme HpyF3I (ThermoFisher, FD1884). After 40 cycles of PCR at 60°C annealing, samples were digested for one hour using specified restriction enzyme. Digested PCR products were resolved using a 1.5% Ultrapure agarose (Invitrogen) and 1.5% Metaphor agarose (Lonza) gel. Genotyping for the *tp53*^*M214K*^ allele was performed as previously described (Berghmans et al., 2005).

### In situ hybridization (ISH)

For whole mount ISH, embryos treated with PTU (to prevent pigment formation) were collected at specified stages, fixed in 4% paraformaldehyde overnight at 4°C, washed in PBS, and dehydrated in methanol. *ISH* was performed according to standard protocols [48], except hybridization was performed at 65°C, maleic acid buffer (100mM maleic acid, pH 8, 150mM NaCl) was substituted for PBS during antibody incubations with alkaline phosphatase (AP)- conjugated anti-DIG antibodies (Roche, 11093274910), and BM Purple was used to develop the chromogenic reaction (Roche, 1442074). For fluorescent ISH in oocytes, whole ovaries were fixed as described above and the same ISH protocol was used until the point of chromogenic detection, at which point Fast Red Tablets (Roche, 11496549001) dissolved in 2 ml 0.1M Tris-HCl was used for the fluorescent detection of AP enzyme activity. To generate antisense probes for *rbpms2* transcripts, a linear template with T7 promoter was amplified from *pcs2-rbpms2a* and *pcs2-rbpms2b* plasmids using the primers:

5’-CAAACGCTGCGTCTGGAGT-3’ (*rbpms2a F*),

5’-TAATACGACTCACTATAGGGCATGTCTCCACCTTTCA-3’ (*rbpms2a T7 R*), and

5’-GCTAAGGCCAACACGAAGAT-3’ (*rbpms2b F*),

5’-TAATACGACTCACTATAGGGCACTGGGCTACACTTC-3’ (*rbpms2b T7 R*).

For *rbpms1* sense and antisense probes, *rbpms1* amplicon (see RT-PCR for primers) was TOPO-cloned into pCR2.1 (ThermoFisher, K450001**),** and plasmids were linearized with HindIII (ThermoFisher, FD0505**).** Probes were *in vitro* transcribed with digoxigenin-UTP labeling kit (Roche, 11175025910). Whole-mount ISH of embryos was imaged using an Olympus SZ61 dissecting microscope with a high- resolution digital camera (model S97809, Olympus America) and Picture Frame 2.0 software.

### Molecular cloning

Constructs used for transient expression assays and co-immunoprecipitation assays were made using the Gateway Recombination LRII Cloning Enzyme Mix (Invitrogen, 11791) to insert the coding sequence of wild-type or mutant *rbpms2a* or *rbpms2b* into the destination vectors pCS-GFP Dest (Addgene Plasmid #13071) or pCS-MT Dest (Addgene Plasmid #13070) [49, 50]. The coding sequences of *rbpms2a* and *rbpms2b* were amplified using Easy-A High Fidelity Taq polymerase (600400, Agilent) and the PCR fragments were TOPO cloned into pCR8/GW/TOPO (K250020, Invitrogen). Wild-type *rbpms2a* and *rbpms2b* were PCR-amplified from AB strain ovary cDNA and sequenced, while the mutant alleles *rbpms2a*^*ae30*^, *rbpms2b*^*ae32*^ and *rbpms2b*^*sa9329*^ were PCR-amplified from cDNA from 4-cell stage maternal-zygotic (MZ) mutant embryos and sequenced, using the primers:

5’- ATGAGTCTGAAGTCAGATTCAGAGAC-3’ (*rbpms2a ATG F*) and

5’- TTAACAGAACTGACGTGATTTCC-3’ (*rbpms2a stop R*), or

5’- ATGAGTGTCAAGTCCGACTC-3’ (*rbpms2b ATG F*) and

5’- TTAACAGAACTGTCGGGATTTCC-3’ (*rbpms2b stop R*).

The constructs used to generate stable transgenic lines (*Tol2-cmcl2GFP-bucP-mApple-rbpms2-3’UTR* and *Tol2-cmcl2GFP-bucP-mApple-rbpms2bΔRNP-3’UTR*) were made using multiple-fragment cloning with the Gateway Recombination LRII+ Cloning Enzyme Mix (Invitrogen, 12538120) to insert the coding sequence and 3’UTR of wild-type *rbpms2b* into the Tol-2 destination vector pDestTol2CG2 (Tol2 kit v1.2 #395) behind the *bucky ball* promoter (p5E-bucP) and mApple RFP sequence (pME-mApple, Tol2 kit v2.0 #763) [14, 49]. The *rbpms2b* coding and 3’UTR sequence was TOPO cloned using amplified products from ovary cDNA into pCR8 as described above using the same F primer (*rbpms2b ATG F*) and 5’-GCATTCCAATTTTAATACTATCATACAGTAGTTTCTTT-3’ (*rbpms2b 3’UTR R*). *pCS-GFP-rbpms2bΔRNP1* and *Tol2-buc-mApple-rbpms2bΔRNP-3’UTR* constructs were made using the QuikChange II Site-Directed Mutagenesis Kit (Agilent, 200523) and mutagenesis primers:

5’-ATCAAGCTAACTTCAAAGGACAGTCGTTCTGGCGCT-3’ (*rbpms2bΔRNP1 F*), and

5’-AGCGCCAGAACGACTGTCCTTTGAAGTTAGCTTGAT-3’ (*rbpms2bΔRNP1 R*). The *rbpms2bΔCterm* constructs were made from *pcs2-MT-rbpms2b* using the QuikChange II Site-Directed Mutagenesis Kit (Agilent, 200523) and mutagenesis primers 5’-GGACTCGTCCCAGCCTGGATTAAGATTCGAGCCTCTAGAAC-3’ (*rbpms2bΔCterm* F) and 5’-GTTCTAGAGGCTCGAATCTTAATCCAGGCTGGGACGAGTCC-3’.

### Localization assays in zebrafish somatic cells and PGCs

For assays of localization in somatic cells and PGCs, *GFP-Rbpms2* and *RFPnos3UTR* [51] plasmids were linearized and then transcribed using the mMessage mMACHINE SP6 Transcription Kit (AM1340, Invitrogen). For *GFP-rbpms2* and *RFPnos3UTR* plasmids, 200pg of RNA was injected into 1-cell embryos. For analysis of somatic cells, injected embryos were fixed at sphere stage in 4%PFA, and for PGC analysis embryos were fixed at 30hpf. Antibody staining was performed as indicated below in the *Immunofluorescence section*.

### Immunofluorescence (IF)

For whole-mount IF of embryos or ovaries, tissue was fixed in 4% paraformaldehyde overnight at 4°C, dehydrated in MeOH, and placed at—20°C. Two anti-Rbpms2 antibodies were used in this study, mαRbpms2 (Abcam, ab169394) a mouse polyclonal raised to full-length human protein (amino acids 1–209 NP_919248), and rαRbpms2 (abcam, ab170777) a rabbit polyclonal antibody raised to a peptide within amino acids 120–149 were diluted at 1:500, Anti-Bucky ball y1165 at 1:500 [14], and anti-GFP antibody (Invitrogen, A10262) was used at 1:500. Chicken anti-Vasa antibody was a gift of Bruce W. Draper and used at 1:1000 dilution. Rabbit anti-ACF7/Macf1 was used at 1:1000 [52]. Rabbit anti-DCP2 (Novus Biologicals, NBP2-16109) and rabbit anti- TIAL-1 (Novus Biologicals, NBP1-79932) were used at1:2000 as in [29]. Alexafluor488, Alexafluor568, CY3, C5 (Molecular Probes) secondary antibodies were diluted at 1:500. Images were acquired using a Zeiss Axio Observer inverted microscope equipped with Apotome or ApotomeII and a CCD camera, or Zeiss Live DuoScan (line-scanning) Confocal. Images were processed in ImageJ/FIJI, Adobe Photoshop and Adobe Illustrator.

### PGC quantification

In zebrafish, Vasa protein localizes in a perinuclear ring of staining around each PGC nucleus that can be used much like a nuclear marker to identify and count individual cells. Z-series image stacks of one lateral half of embryonic gonads were obtained using a Zeiss Live DuoScan (line-scanning) confocal microscope, and cells in the stack were manually counted by analyzing the slices and nuclear morphology comprising each Z-stack with ImageJ/FIJI.

### Transfections and Co-Immunoprecipitation (Co-IP)

Co-IPs were performed as previously described (Heim et al., 2014). Briefly, sub-confluent HEK293 cells (1×10^6^) were transfected with 3 μg pCS3-MT-Rbpms1/2a/2b, or the specified pCS3-MT-Rbpms2b mutation/truncation, and 3 μg pCS3-GFP-Buc or pCS3-GFP (control) with 3 to 1 ratio of polyethylenimine:DNA, overnight. IP was performed on supernatant from cell lysates (including mRNA) with 1 mg of anti-GFP 3E6 antibody (A11120, Invitrogen) and Protein G magnetic beads (S1430, NEB). Precipitated proteins were separated by SDS-PAGE, and transferred to ImmobilonP (Millipore). Membranes were blotted using 1:2000 anti-Myc (9E10, Santa Cruz) or 1:2000 anti-GFP (11814460001, Roche), washed in TBST, then stained with 1:25,000 goat anti-mouse HRP (12–349, Millipore) prior to ECL detection (GE Healthcare). To visualize granules in HEK293 cells, the same transfection protocol was used with pCS3-GFP-Rbpms wild type and mutant constructs, and the cells were plated onto cover-slipped dishes and photographed live.

### Transmission electron microscopy

For semithin and ultrathin sections, samples were fixed with 2.5% glutaraldehyde, 2% paraformaldehyde in 0.1 M sodium cacodylate buffer, postfixed with 1% osmium tetroxide followed by 2% uranyl acetate, dehydrated through a graded series of ethanol and embedded in LX112 resin (LADD Research Industries, Burlington VT). Ultrathin sections were cut on a Reichert Ultracut UCT, stained with uranyl acetate followed by lead citrate and viewed on a JEOL 1200EX transmission electron microscope at 80kv.

### Histology

Haematoxylin and Eosin (H&E) staining was performed as previously described (Heim et al., 2014). Briefly, tissue was fixed in 4% paraformaldehyde overnight at 4°C, dehydrated in MeOH, and placed at—20°C. After paraffin embedding and sectioning onto slides, tissue was deparaffinized, stained with H&E, coated with Permount solution (Fisher Scientific), coverslipped, and imaged using an AxioSkop2 microscope and AxioCam CCD camera.

### RT-PCR

Total RNA was extracted from pooled embryos (n = 20-30/stage) or pools of 2–3 adult organs using Trizol (Life Technologies, 15596). cDNA was prepared with SuperScript III Reverse Transcription Kit (Life Technologies, 18080–051). RT-PCR was performed using the primers 5’-ATTCACCTCTAAACAGCCGGT-3’ and 5’-AGGCTAGGCTAATCATTACACTG-3’ for *rbpms1*, 5’-ATGCGTTAAATGGCATCCGC-3’ and 5’-GTCCTCAGCATCTCTACCGC-3’ for *rbpms2a*, 5’-CAACGCATCTGAGCATGAAG-3’ and 5’-GATCCAGTCGCACTTTAAGGA-3’ for *rbpms2b*. The primers for *ef1α*, *vasa and cyp19a1a are as described in* [14].

### Phylogram

FASTA protein sequences corresponding to the longest known alternative transcript for each of the zebrafish *rbpms* genes and Xenopus *hermes* were obtained from the Ensembl website (www.ensembl.org) and analyzed using ClustalW for multiple alignments and JalView software to visualize. Phylogeny tree is calculated for neighbor joining using percent identity. Aligned sequences include *rbpms (*herein *rbpms1)* (ENSDART00000127288), *rbpms2a* (20ENSDART00000067514), *rbpms2b* (ENSDART00000006619) and *xhermes* (ENSXETT00000024635).

## Supporting information

S1 Supporting Information File containing all supporting figures. (PDF)
