# Correction: *rbpms2* functions in Balbiani body architecture and ovary fate

**DOI:** 10.1371/journal.pgen.1007768

**Published:** 2018-10-30

**Authors:** Odelya H. Kaufman, KathyAnn Lee, Manon Martin, Sophie Rothhämel, Florence L. Marlow

The images for Figs 5 and 6 are incorrectly switched. The image that appears as Fig 5 should be Fig 6, and the image that appears as Fig 6 should be Fig 5. The figure captions appear in the correct order.

**Fig 5:**
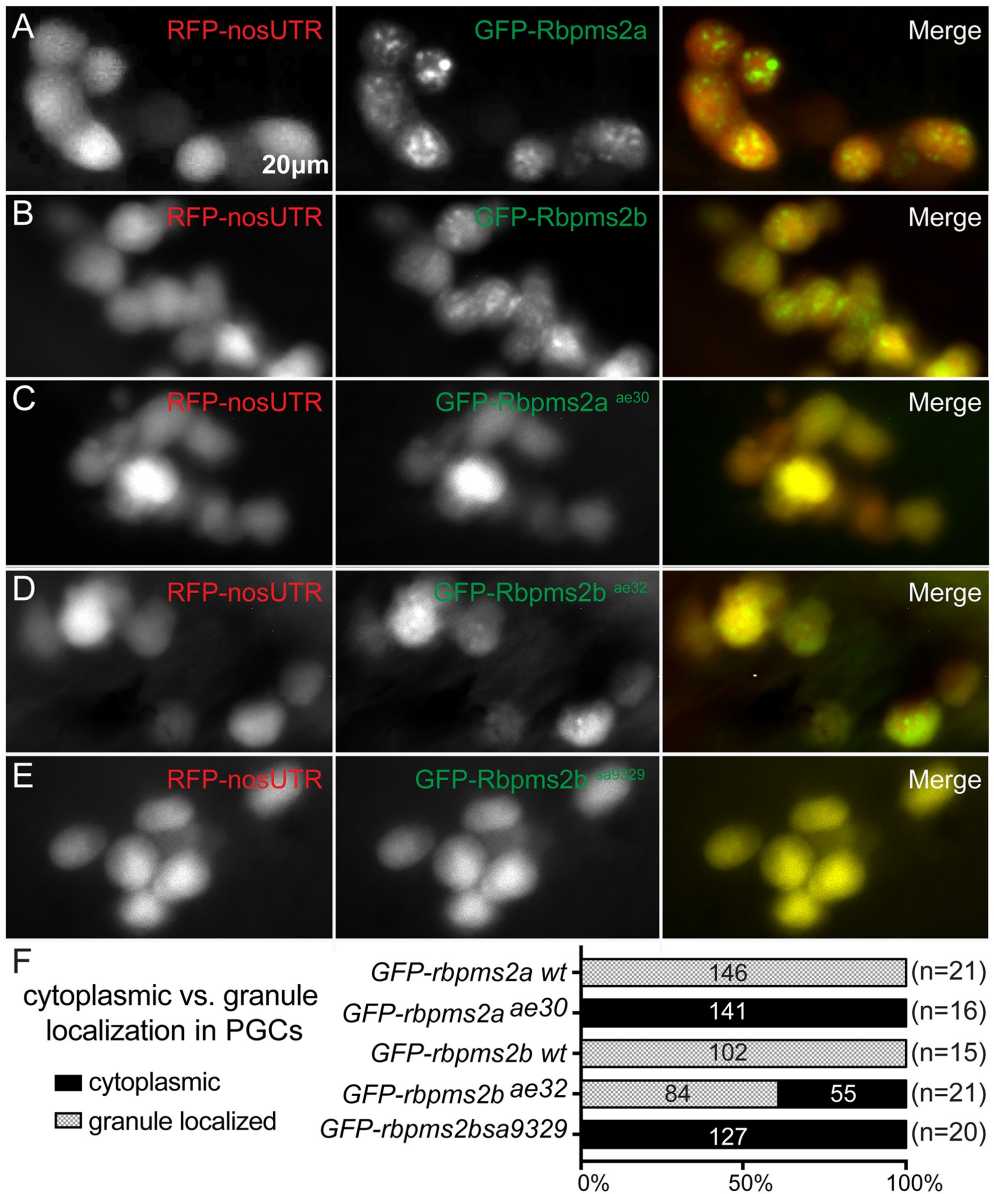
*rbpms2* mutant allele stability and localization activity in germ cells. (A-B) Wild-type GFP-Rbpms2a/b localize to germ granules of PGCs. PGCs are labeled with RFP-*nos3’utr* at 30hpf. (C-E) Like wild-type Rbpms2a and Rbpms2b, GFP-Rbpms2a^ae30^ (C), GFP-Rbpms2b^sa9329^ (D), and GFP-Rbpms2b^ae32^ (E) are all enriched in germ cells. However, GFP-Rbpms2a^ae30^ and GFP-Rbpms2b^sa9329^ are not localized to granules; whereas, GFP-Rbpms2b^ae32^ is partially localized to germ granules. (F) Graph depicting percentage of granule vs. cytoplasmic localization of *GFP-rbpms2* alleles in PGCs. Numbers indicated in parentheses near allele name are number of embryos analyzed, whereas numbers in the right three columns represent numbers of PGCs.

**Fig 6:**
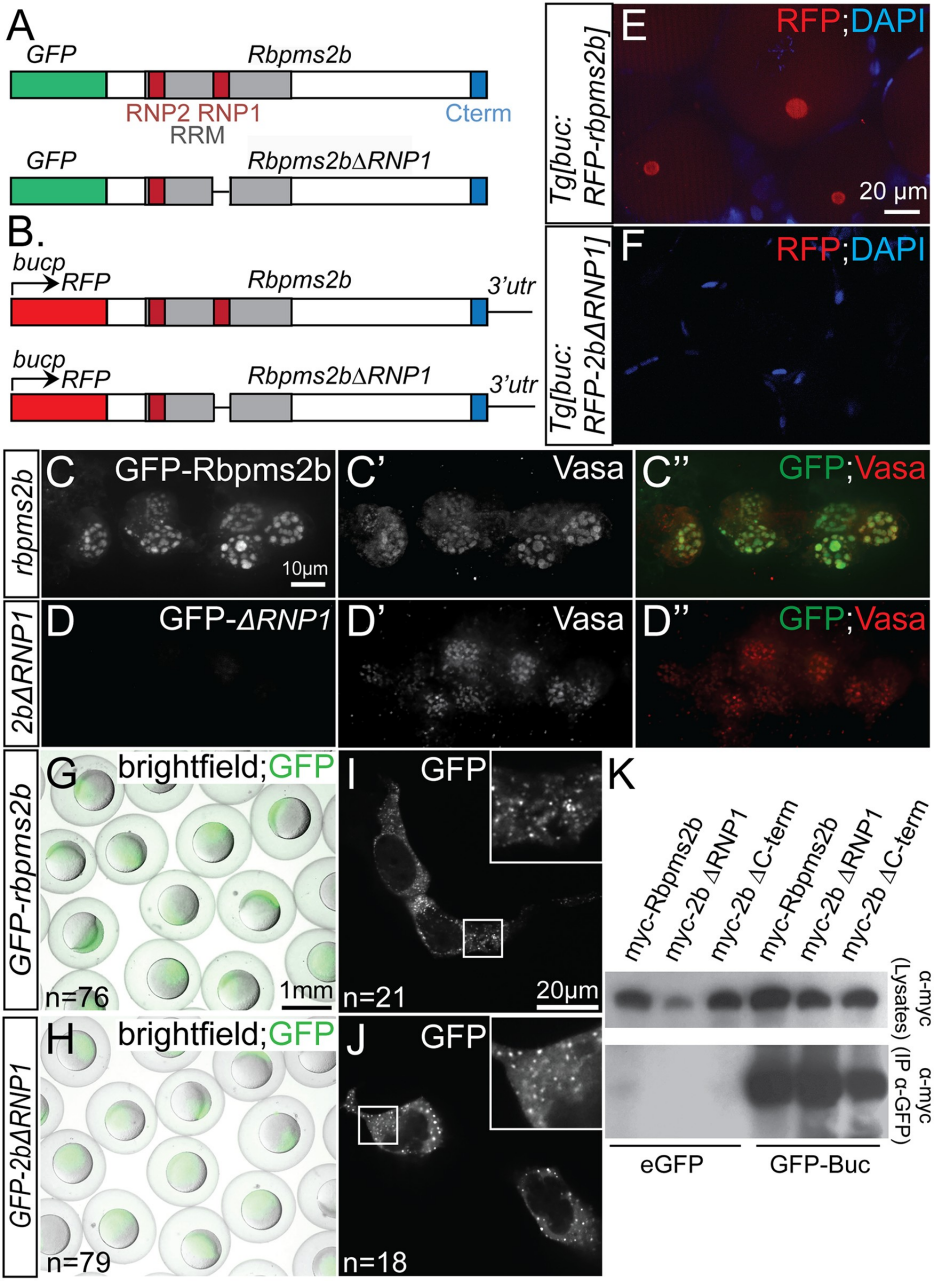
RNA-binding domain is required for Rbpms2 localization in germ cells. (A) Schematic representing constructs used in granule localization assays. (B) Schematic representing transgenic constructs used to express Rbpms2b in oocytes. (C-C”) Wild-type GFP-Rbpms2b localizes with Vasa in germ granules of PGCs. (D-D”) GFP-Rbpms2bΔRNP1, a deletion of 7 residues within the RRM domain, does not localize to germ granules. (E-F) RFP-Rbpms2b expressed in transgenic Tg[buc:RFP-rbpms2b] ovaries localizes to the Bb (E), while RFP-Rbpms2bΔRNP1 does not (F). (G-J) GFP-Rbpms2b and GFP-Rbpms2bΔRNP1 are stably expressed in gastrula stage embryos (G,H), as well as the granules of HEK293 cells (I, J). (K) GFP immunoprecipitation of Myc- tagged Rbpms2b, Rbpms2bΔRNP1, and Rbpms2bΔC-terminus, a deletion of the last 7 residues within the conserved C-term, demonstrates that the RNP1 domain or conserved C-terminus is not required for interaction with GFP-Bucky ball.

There is an error in Fig 9. The outline around the mitochondria adjacent to the nucleus has shifted upwards in Fig 9D. Please see the corrected Fig 9 here.

**Fig 9:**
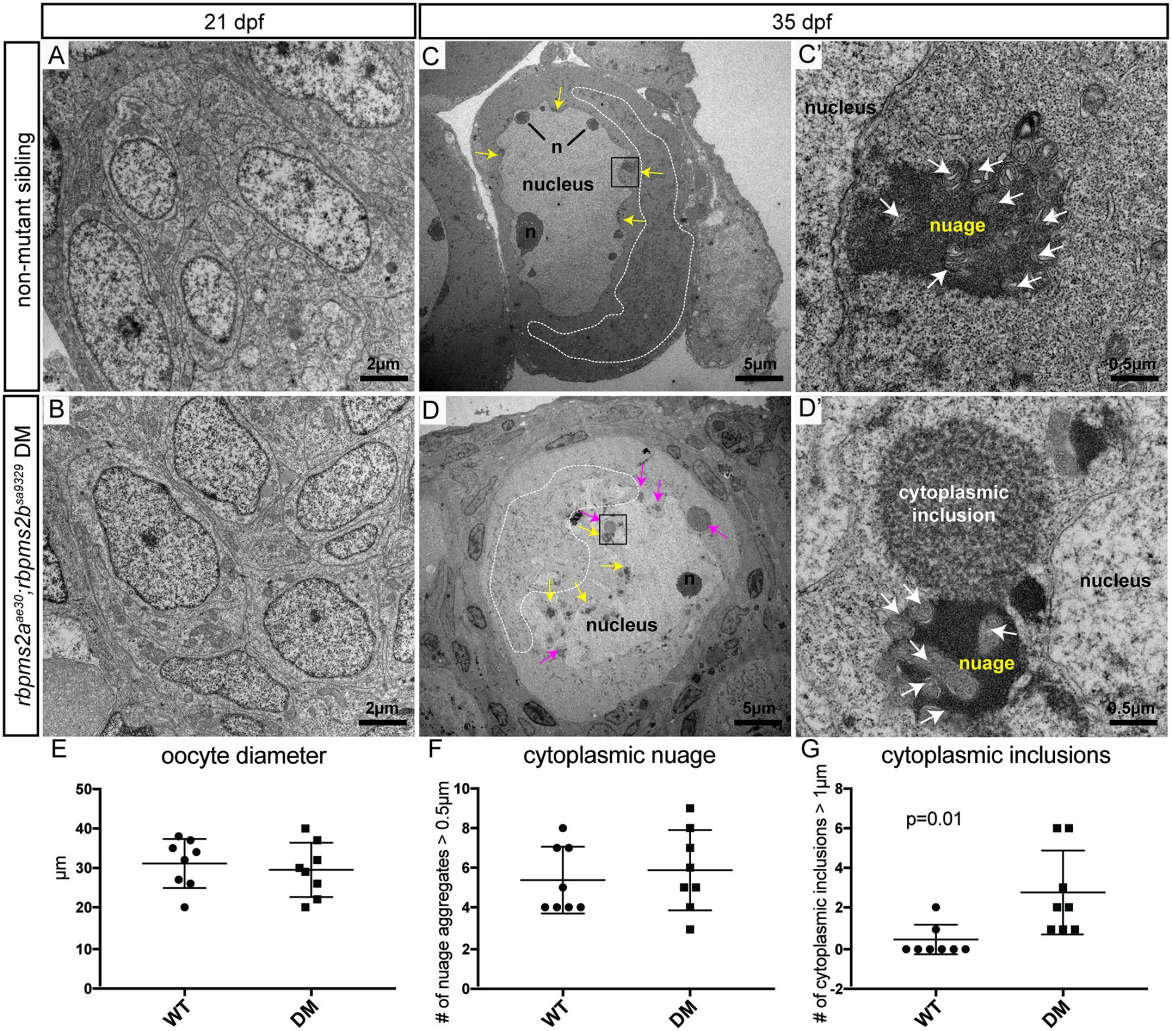
*rbpms2* mutant oocytes contain atypical cytoplasmic inclusions. (A-B) Comparable clusters of gonocytes from d21 non-mutant siblings (A), and *rbpms2* double mutants (B). (C,D) Oocytes from d35 non-mutant siblings (C), and *rbpms2* double mutants (D). Marked features include: n = nucleoli, white dotted line outlining areas of mitochondrial enrichment, yellow arrows indicating nuage accumulations, pink arrows indicating atypical cytoplasmic inclusions. Areas marked in a black box in (C, D) are magnified in (C’,D’). (C’, D’) High magnification images of nuage with associated mitochondria (white arrows) in non-mutant oocyte (C’), and juxtaposed nuage and atypical cytoplasmic inclusion in *rbpms2* mutant oocyte (D’). (E-G) Oocytes of comparable diameter (E) were used to quantify and compare number of nuage aggregates over 0.5μm (F), and number of cytoplasmic inclusions figover 1μm (G). Significance was analyzed with unpaired t-test.
