# Supplementary material for: rbpms2 functions in Balbiani body architecture and ovary fate
Source: PLoS Genet. 2018 Jul 5;14(7):e1007489. doi: 10.1371/journal.pgen.1007489 (PMC6049948; doi:10.1371/journal.pgen.1007489)
Supplement: S1 Supporting Information — (PDF) [file pgen.1007489.s001.pdf]

Supplementary Figure Legends

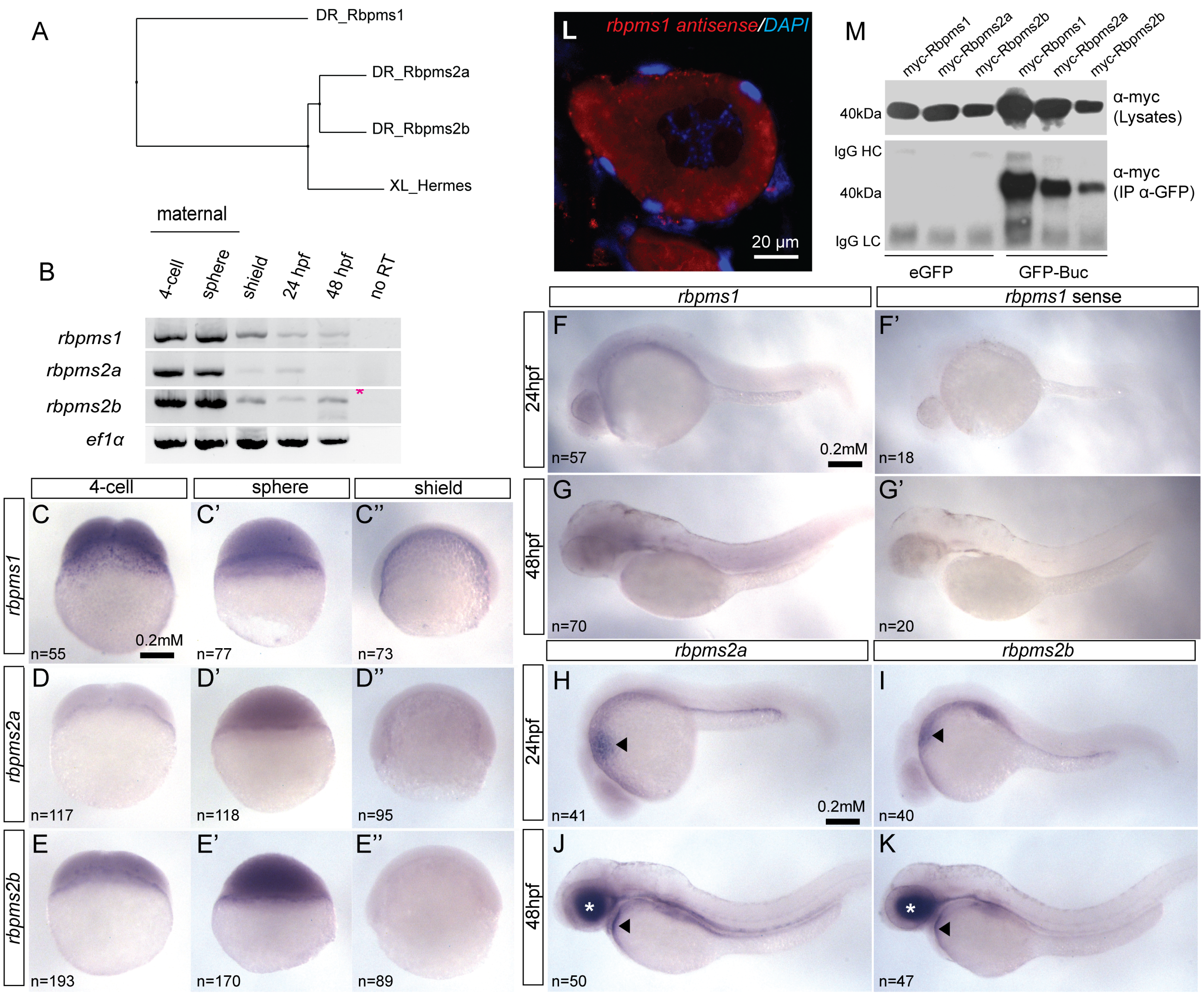

Supplementary Figure 1. Conserved expression of *Xhermes* homologs in zebrafish.

(A) Phylogenetic tree clustered according to protein sequence similarity between *Xenopus* Hermes and zebrafish Hermes homologs Rbpms1, Rbpms2a, and Rbpms2b. (B) RT-PCR on maternal and zygotic stages of zebrafish embryonic development demonstrate all three RNAs are abundant as maternal transcripts. (C-E'') Lateral views of whole-mount *in situ* hybridization (ISH) for *rbpms1*, *rbpms2a* and *rbpms2b* in 4-cell, sphere, and shield- stage embryos. Lateral views of 24 hpf ISH for *rbpms1* (F), and sense probe control (F'). Lateral views of 48 hpf ISH for *rbpms1* (G), and sense probe control (G'). Lateral views of 24 hpf embryos shows similar *rbpms2a* (H) and *rbpms2b* (I) ISH staining in heart primordium (arrow heads).

Lateral view of 48 hpf embryos reveals similar *rbpms2a* (J) and *rbpms2b* (K) ISH staining pattern in the heart (arrow head) and retina (white asterisk). (L) Fluorescent *in situ* hybridization displaying unlocalized *rbpms1* transcripts in primary zebrafish oocyte. (M) GFP immunoprecipitation of Myc- tagged Rbpms1, Rbpms2a and Rbpms2b demonstrates that that all three proteins can interact with GFP-Bucky ball *in vitro*.

A

```
Rbpms2a     1 MSL KSDSETNTS VSLEEEVRTLFVSGLPVDIKPRELYLLFRPFKGYEGSLIKLTSKQPVG
Rbpms2b     1 MSVKSDSEPNNNVSLEEEVRTLFVSGLPTDIKPRELYLLFRPFKGYEGSLIKLTSKQPVG

Rbpms2a    61 FVTFDSRSGAEAAKNALNG I RFDPESPQTLRLEFAKANTKMAKSKLMATPNPSNLHPALG
Rbpms2b    61 FVTFDSRSGAEAAKNALNGVRFDPENPQTLRLEFAKANTKMAKSKLMGTPNPTNI HPALG

Rbpms2a   121 AHFIARDPYDLTGAALIPASPEAWAPYPLYT TELTPGL PHAAFTYPAAAAAAAA -LHAQM
Rbpms2b   121 AHFIARDPYDLTGAALIPASPEAWSPYPLYTPELSPGLPHTAFTYPAAAAAAAAALHAQM

Rbpms2a   180 RWYPSPSESSQPGWKSRQFC
Rbpms2b   181 RWYPSTSDSSQPGWKSRQFC
```

B

rbpms2a wt (exon 5 partial)
5'- AGAGTAAACTGATGGCCACGCCGAACCCTTCCAACCTGCACCCTGCT-3'

rbpms2a ^{ae27} (in-frame 27bp deletion)
5'-AGAGTAA.. CCTGCACCCTGCT-3'

rbpms2a ^{ae30} (15p deletion, 17bp insertion)
5'- AGAGTAAACTGAGAGTAAACTGAGAGCAGCTTCCAACCTGCACCCTGCT-3'

C

rbpms2b wt (exon 5 partial)
5'-AGAGTAAGCTGATGGGCACTCCGAATATCACAAATATCCACCCAGCT-3'

rbpms2b ^{ae32} (20bp insertion)
5'-AGAGTAAGCTGATGGGCACTCCGAATATCCACCCAGATATCCACCCAGCACAAATATCCACCCAGCT-3'

rbpms2b wt (exon3-4 boundary)
ATCAAGCTAACTTCAAAGCAGGTGAGACATTGTTAGACATTACA

rbpms2b ^{sa9329} (splice site mutation)
ATCAAGCTAACTTCAAAGCAGGAGAGACATTGTTAGACATTACA

Supplementary Figure 2. Rbpms2 protein alignments and genomic loci of mutations in *rbpms* family members.

(A) Rbpms2a and Rbpms2b protein alignments with disparate residues shown in blue font. Shaded grey domain is the RNA Recognition Motif (RRM), shaded red domains are the Ribonuclear Particle (RNP)

domains, and shaded blue denotes the species conserved C-terminal domain. (B) *rbpms2a* genomic locus showing the wild-type allele (top) with the Crispr target site in red, the *rbpms2a*^{ae27} allele with deleted bases represented by dashes, and the *rbpms2a*^{ae30} allele with insertion represented by purple base pairs. (C) *rbpms2b* genomic locus showing the fifth exon of the wild-type allele (top) with the Crispr target site in red, the *rbpms2b*^{ae32} allele with insertion represented by purple bases. Below that is the 3rd exon/intron boundary (red) of wild-type *rbpms2b*, and the Sanger allele *rbpms2b*^{sa9329} splice-site mutation (purple).

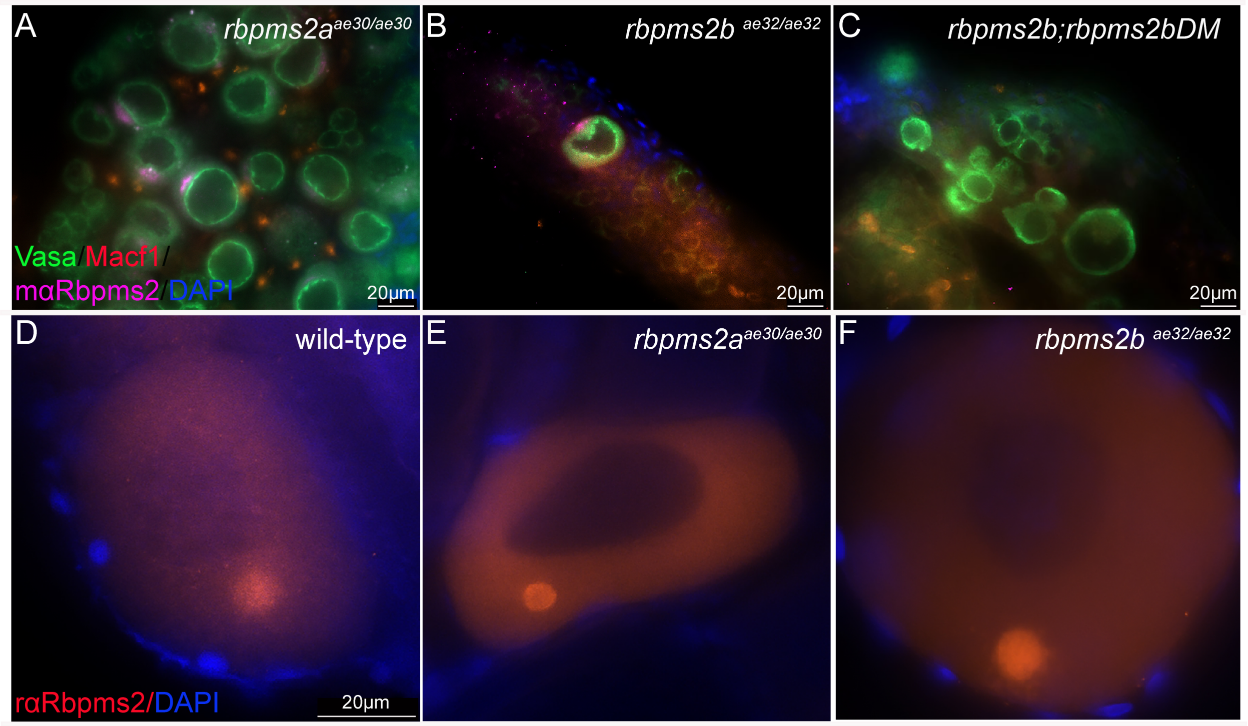

Supplementary Figure 3. Rbpms2, Vasa and Macf1/Mgn in *rbpms2* mutant ovaries. Antibody staining for Rbpms2 (magenta), Vasa (green), MacF1 (red) and DAPI (blue) proteins in juvenile ovaries (d35) of (A) single mutant for *rbpm2a* (B) single mutant for *rbpm2b,* and (C) double mutant. (D-F) rRbpms2 antibody staining (red) and DAPI (blue) in adult ovary of (D) WT, (E) single mutant for *rbpm2a* (F) single mutant for *rbpm2b*. n=5 gonads per genotype.

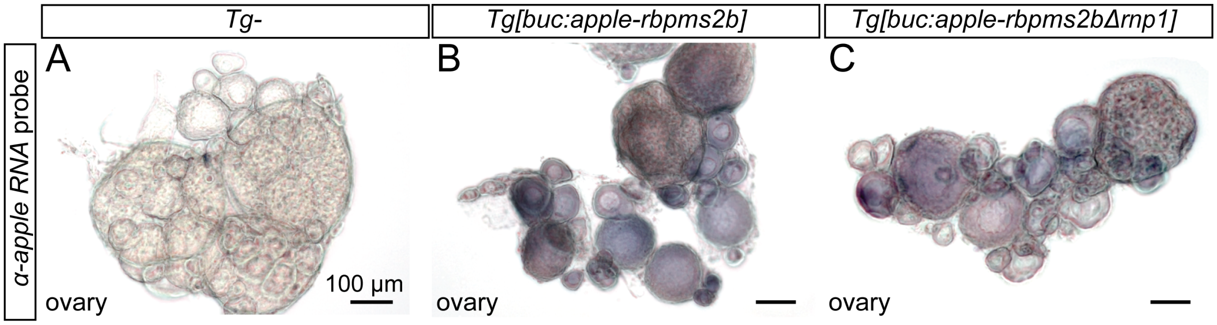

Supplementary Figure 4. RNA transcripts in *rbpms2* transgenic ovaries. Whole-mount *in situ* hybridization on ovary fragments was performed using anti-sense *apple (RFP)* probe and colorimetric probe detection. (A) Control transgenic negative (wild-type) ovaries demonstrate the baseline staining for this probe. (B,C) Ovaries from *Tg[buc:RFP-rbpms2b]* and *Tg[buc:RFP-rbpms2bΔRNP1*] both display alkaline phosphatase staining, indicating the presence of *apple-rbpms2b* (B) and *apple-rbpms2bΔRNP1* (C) transcripts in oocytes.

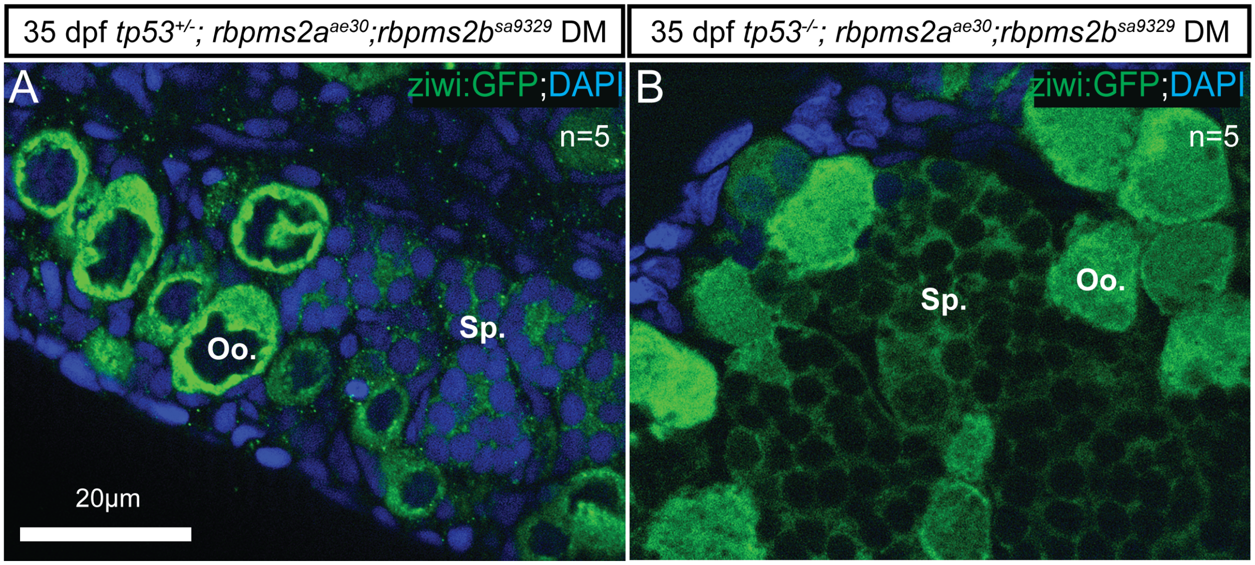

Supplementary Figure 5. Tp53 mutation does not modify *rbpms2* DM phenotype. Transgenic germ cell marker *ziwi:GFP* allows visualization of comparable intersex germ cell phenotype in d35 gonads of *tp53* $^{M214K/+}$ *;rbpms2DM* (A) and *tp53* $^{M214K/\,M214K}$*;rbpms2DM* (B). Oo. = oocyte-like cell; Sp.= spermatocyte-like cells.

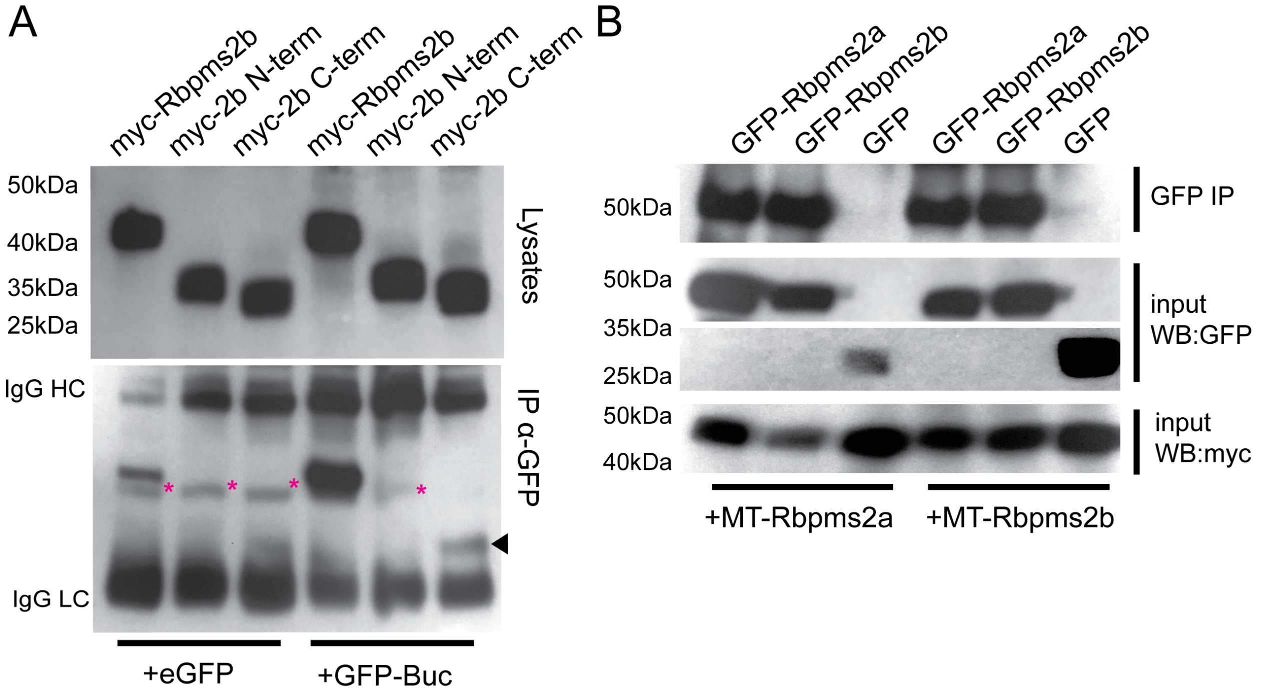

Supplementary Figure 6. Rbpms2 interacts with Bucky ball through its C-terminus, and Rbpms2a and 2b can form heterodimers. (A) GFP immunoprecipitation of Myc- tagged Rbpms2b, the N terminus of Rbpms2b (residues 1-100) and the C-terminus of Rbpms2b (residues 101-200) demonstrates that the C-terminal half is required and sufficient for interaction with Bucky ball. Pink asterisk: the weak lower band is a nonspecific product, black arrowhead points at pull down of Rbpms2b C-terminus. (B) GFP immunoprecipitation of Myc- tagged Rbpms2a and Rbpms2b demonstrates that that both proteins can homodimerize and Rbpms2a can heterodimerize with Rbpms2b.
